# Application of a Substrate-Mediated Selection with c-Src Tyrosine Kinase to a DNA-Encoded Chemical Library

**DOI:** 10.3390/molecules24152764

**Published:** 2019-07-30

**Authors:** Dongwook Kim, Yixing Sun, Dan Xie, Kyle E. Denton, Hao Chen, Hang Lin, Michael K. Wendt, Carol Beth Post, Casey J. Krusemark

**Affiliations:** 1 Department of Medicinal Chemistry and Molecular Pharmacology, Purdue University Center for Cancer Research, Purdue University, West Lafayette, IN 47907, USA; 2 Dencoda, LLC, West Lafayette, IN 47906, USA; 3 X-Chem Pharmaceuticals, Waltham, MA 02453, USA

**Keywords:** DNA-encoded chemical library, protein tyrosine kinases, substrate-mediated selection, c-Src

## Abstract

As aberrant activity of protein kinases is observed in many disease states, these enzymes are common targets for therapeutics and detection of activity levels. The development of non-natural protein kinase substrates offers an approach to protein substrate competitive inhibitors, a class of kinase inhibitors with promise for improved specificity. Also, kinase activity detection approaches would benefit from substrates with improved activity and specificity. Here, we apply a substrate-mediated selection to a peptidomimetic DNA-encoded chemical library for enrichment of molecules that can be phosphorylated by the protein tyrosine kinase, c-Src. Several substrates were identified and characterized for activity. A lead compound (**SrcDEL10**) showed both the ability to serve as a substrate and to promote ATP hydrolysis by the kinase. In inhibition assays, compounds displayed IC_50′_s ranging from of 8–100 µM. NMR analysis of **SrcDEL10** bound to the c-Src:ATP complex was conducted to characterize the binding mode. An ester derivative of the lead compound demonstrated cellular activity with inhibition of Src-dependent signaling in cell culture. Together, the results show the potential for substrate-mediated selections of DNA-encoded libraries to discover molecules with functions other than simple protein binding and offer a new discovery method for development of synthetic tyrosine kinase substrates.

## 1. Introduction

DNA-encoded chemical libraries (DELs) have recently become a widespread approach for the discovery of new protein ligands [1,2]. DELs offer advantages in cost, throughput, and access to greater chemical space over conventional de novo discovery by high throughput screening. Discovery of several new inhibitors to many classes of therapeutically relevant protein targets has now been reported [3]. These include molecules currently in clinical trials.

The predominant approach to ligand discovery from DELs is an affinity-mediated selection assay, whereby DNA-linked ligands are enriched from the library through binding to a protein target immobilized on a solid support [4]. While successful in many cases, these assays can suffer from drawbacks, such as background binding to the support matrix, limited control of protein concentration, and the potential for loss of protein activity with immobilization. Also, by design, affinity-mediated selections enrich simply for the function of binding. While seldom reported or investigated thoroughly, such selections can result in so called “innocent binders”, which do bind the protein but do not inhibit the desired protein activity. Thus, an oft-cited goal for DEL assays is to develop selections to yield molecules with functions beyond simple protein binding, with the hope that all hit molecules will inhibit a desired activity of the protein target. One approach to this is to perform parallel selections in the presence or absence of known protein binders (inhibitors or substrates) to illuminate the binding mode of hit ligands [5]. Another approach is the release of small molecules from bead-bound DELs within confined droplets for colorimetric inhibition assays in microfluidic devices [6]. Yet another approach is a substrate-based selection, which can enrich molecules on the basis of enzymatic turnover [7]. Substrate selections have seen application in both phage display and mRNA display [8,9], but have seen limited application with DELs [10].

Here, we employ a substrate-mediated selection for selecting phospho-accepting substrates of the tyrosine kinase c-Src, that could then serve as protein substrate competitive inhibitors. Not only should such a selection yield hits that bind specifically at the protein substrate site, but it should also be less prone to non-specific interactions than could enrich molecules in a typical affinity-mediated selection. A substrate-mediated selection also has greater potential for sensitivity in enriching low affinity hits as it can take advantage of enzymatic turnover (much like an ELISA), and therefore it has a lower requirement for specific activity of the protein target, which can be problematic for protein targets with poor stability.

Protein kinases are well-validated drug targets and the second largest drug target class [11]. Yet, there remains a critical need for kinase inhibitors that are potent, highly specific, and not susceptible to drug resistance mutations. Nearly all reported kinase inhibitors and the vast majority of the 37 clinically-approved therapeutics target the ATP binding site [12]. With ~518 protein kinases utilizing ATP as a substrate, achieving specific inhibition with ATP competitive inhibitors is a challenge [13].

Strategies that exploit less-conserved residues adjacent to the ATP-binding site have improved selectivity, yet arising mutations to these residues can abolish drug binding with minimal effects on kinase enzymatic function. For example, a “gatekeeper” mutation (T315I) in Bcr-Abl leads to imatinib resistance but shows little effect on the binding of ATP [11]. The development of such resistance mutations is a major problem with kinase inhibitors used in cancer treatment. Rather than targeting inhibitors to areas distal from the active site to achieve selectivity, targeting sites most critical to kinase activity will more likely yield inhibitors recalcitrant to resistance, as such mutants are likely to be non-functional.

In addition, ATP-competitive inhibitors have a very high requirement for affinity to achieve cellular activity due to the typical K_m_ values for ATP being low to mid- micromolar and cellular ATP levels being low millimolar [14]. The potency required for efficacy with ligands targeting the protein substrate site is much lower, since substrates in cells are generally present at concentrations near or below their K_m_ [15].

While kinase substrate interactions are often mediated by additional proteins in vivo, kinases possess a great deal of “naked” substrate specificity. Optimal peptide substrates, determined with purified kinases in in vitro assays, vary in sequence considerably among kinases [16]. Kinase residues in the substrate binding site are much less conserved than the ATP site [17]. While in vitro studies with kinases have shown overlap in peptide substrate sequence, there are clear, distinct substrate preferences, even among highly related kinases, which demonstrates potential for differentiation [18]. Generally, protein tyrosine kinases prefer peptide substrates with multiple negatively charged amino acids [19]. Optimization of natural peptide substrates for several non-receptor tyrosine kinases found an optimal sequence for c-Src of EEEIYGIFG (k_cat_ = 2.9 s^−1^, K_m_ = 14 µM). Prior work from Lam and coworkers generated efficient substrates for c-Src that lack acidic residues completely, which demonstrates high plasticity in substrate recognition. These include sequences GIYWHHY (K_m_ = 21 µM) and YIYGSFK (K_m_ = 55 µM) [20]. These peptides have been converted into pseudosubstrate inhibitors with low µM IC_50′_s [21].

Prior work has demonstrated that even homologous kinases can be differentially inhibited through pharmacological targeting at the protein-substrate site [22]. For example, work with modified peptide substrates of the PKC family generated inhibitors with remarkable potency and selectivity [23]. Work from Soellner and coworkers presented a substrate-based fragment screening approach for discovery of protein substrate competitive ligands [24]. This technique was successful in generating the first substrate competitive inhibitor for c-Src, which demonstrated modest potency (16 μM K_i_), yet excellent selectivity among Src family kinases and activity in cell culture. Additionally, work with Src and DNA-encoded peptide macrocycles yielded inhibitors that exhibited bisubstrate-competitive behavior. Interestingly, these molecules inhibited substrate binding allosterically and showed excellent specificity over other Src family kinases [25].

A wide variety of techniques rely on peptides as protein kinase substrates for detection and profiling of kinase activity in biological samples. Not only are such approaches critical for drug development research [26], but they are also showing the potential utility of kinase activity as a disease biomarker in the ex vivo analysis of patient samples [27]. Examples of such methods include peptide microarrays [28], cell penetrating fluorescent sensors [29,30], and peptide reporters for detection by capillary electrophoresis [31]. Compared to the mass spectrometry-based, kinase enrichment approaches, like multiplexed inhibitor beads [32] and acyl-ATP probes [33], these methods have the advantage that activity is directly measured and not simply kinase abundance. The drawback, however, is that peptide substrates have poor specificity. In the majority of these detection approaches, non-natural substrates could substitute for natural peptide sequences directly. In addition to the potential for greater kinase selectivity, synthetic substrates could have advantages in protease stability and increased activity for improved assay sensitivity.

In this work, we use the c-Src protein tyrosine kinase and a substrate-based selection for discovery from a DNA-encoded library (DEL) of peptidomimetics. We present a DEL designed to target the protein substrate site that contains natural peptides, non-natural peptides, and peptoid-inspired structures. Treatment of the library with ATP and Src kinase followed by an affinity selection using a non-specific phosphotyrosine-binding antibody allowed enrichment of substrate molecules.

## 2. Results

### 2.1. DNA-Encoded Chemical Library

**Scheme 1:**
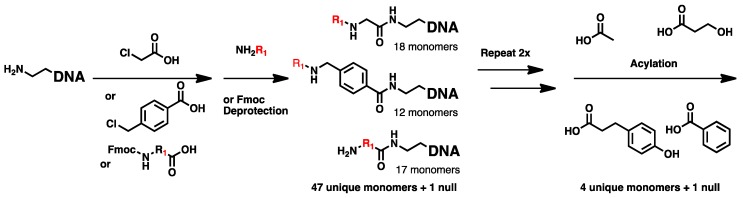
Construction of DNA encoded library with 5.5 × 10^5^ unique members.

A DNA-encoded library was constructed in 4 encoding cycles using the routing-based approach of DNA-programmed combinatorial chemistry [34,35]. The majority of the library diversity was incorporated within the first three cycles yielding 3-mer oligoamides composed of 47 unique monomers, which were acylated in the fourth step with any of 4 carboxylates (Scheme 1). Monomers in the first three steps included amino acids (both natural and non-natural), peptoids (*N*-substituted oligoglycines), and peptoid-inspired *N*-alkyl benzyl monomers [36,37] (Scheme 1). See Tables S1 and S2 for full monomer set. These building blocks gave a full library complexity of 5.5 × 10^5^ unique members. Monomers were selected for the library to maximize both chemical diversity and reaction efficiency. Both fmoc-amino acid peptide chemistry and peptoid synthesis have been demonstrated on DNA and have shown to be quite robust [38,39]. Fmoc-amino acids included showed at least 90% acylation of a DNA-linked amine in rehearsal reactions (Tables S1 and S2) or in previous reports of on-DNA chemistry [38]. Similarly, all amines included were tested in the standard peptoid chemistry and demonstrated greater than 80% conversion in the SN2 reaction of a chloroacetamide on-DNA and at least 50% conversion in the subsequent acylation with chloroacetate. The library was prepared such that each building block was encoded by two different codons with the exception of building blocks for the fourth step, which were singly encoded. The codon sets were kept separate until after translation and then combined to give a two-fold genetic redundancy for each library member.

This library design was selected to bridge the gap in structure from natural peptide substrates towards molecules with more drug-like properties. The tertiary amides of both the peptoid and peptoid-inspired monomers are increasingly hydrophobic compared to the secondary amides of peptides. Peptoids have shown markedly greater permeability than peptides in several studies [40,41]. Additionally, several phenol containing building blocks were included to increase the likelihood of suitable phospho-acceptors. Targeting the protein substrate site with conventional medicinal chemistry approaches has been challenging, and thus it has been sparsely explored [15]. The shallow, exposed, and extended binding site of the kinase substrate protein-protein interaction is not amenable to structure-based design nor is it well suited to binding small, flat heterocycles typical of high throughput screening libraries [42].

As a quality control measure, the library was sequenced before and after translation. The codon abundances were shown to be narrowly distributed and did not change appreciably after translation, indicating that little codon bias occurs in the process of hybridization routing and chemistry (Figure S1). Based on codon abundances in the translated library, gene abundances were approximated for the 2 × 10^5^ genes encoding the initial 3 steps, as previously described [10]. The maximum disparity in gene abundance was 100-fold and the vast majority of the library (95%) was within a 10-fold window (Figure S1c). To further validate both genetic and chemical steps in library assembly, we substituted one of the building blocks at chemical step 3 with a known pharmacophore (an aryl sulfonamide) to carbonic anhydrase II (CAII) and demonstrated enrichment of ligands by DNA sequencing (Figure S2). All of the top 400 enriching molecules contained the pharmacophore. SAR of the enriched molecules was consistent with previous published ligands [43].

### 2.2. Tyrosine Kinase Substrate-Mediated Selection

**Figure 1:**
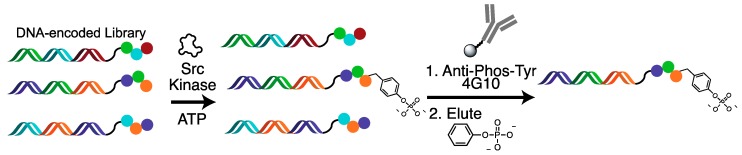
Src kinase substrate selection scheme.

We employed a two-step selection strategy to enrich for molecules that can serve as substrates to c-Src (Figure 1). DNA-encoded molecules are treated with active c-Src kinase and ATP. Phosphorylated molecules are then enriched with a broad specificity anti-phosphotyrosine antibody (4G10 clone) immobilized on Protein G magnetic beads [44]. This approach has been applied in phage display libraries for discovery of natural peptide substrates [45]. A potential drawback of the approach is that certain non-natural substrates may not be recognized by the antibody. Also, as c-Src has been shown to phosphorylate aliphatic oxygens [46], these substrates are unlikely to be enriched by this approach. Given that phosphoprotein and phosphopeptides can be eluted from antibody supports with phenyl phosphate [47], we hypothesized that this antibody contains reasonable affinity for simple phosphorylated phenols. In order to minimize bias in the selection based on variation in affinity for the antibody, antibody beads were used at as high a concentration as possible. Selections employing ATP-γS as a co-substrate with subsequent labeling of phosphothiolates were additionally explored but were met with several problems [7,10]. ATP-γS is a non-natural and poor substrate for c-Src [48]. Commercially available ATP-γS is significantly contaminated with high levels of inhibitory ADP [49]. Additionally, we observed low, but significant, reactivity of DNA with both iodoacetamide biotin and pyridyldisulfide biotin in attempts to selectively label DNA-linked phosphothiolates and were concerned that certain library building blocks may have higher levels of reactivity. Thus, we found using the native ATP substrate with the antibody pulldown to be preferred and a more robust approach.

To validate this selection strategy, we performed test selections using a DNA-linked peptide substrate. We used an optimal peptide substrate (LYNtide) for Lyn tyrosine kinase, a Src family kinase, which showed similar kinetic parameters for Src (k_cat_ = 2.0 s^−1^, K_m_ = 18 µM) [18].

To mimic library selection conditions, a DNA-LYNtide construct was mixed with a control construct at a ratio of 1 to 500. This mixture was treated at 50 nM total DNA concentration with Src kinase and the selection was applied. We observed robust enrichment (2600-fold) and recovery (9%) of the LYNtide construct by qPCR (Figure S3).

**Figure 2:**
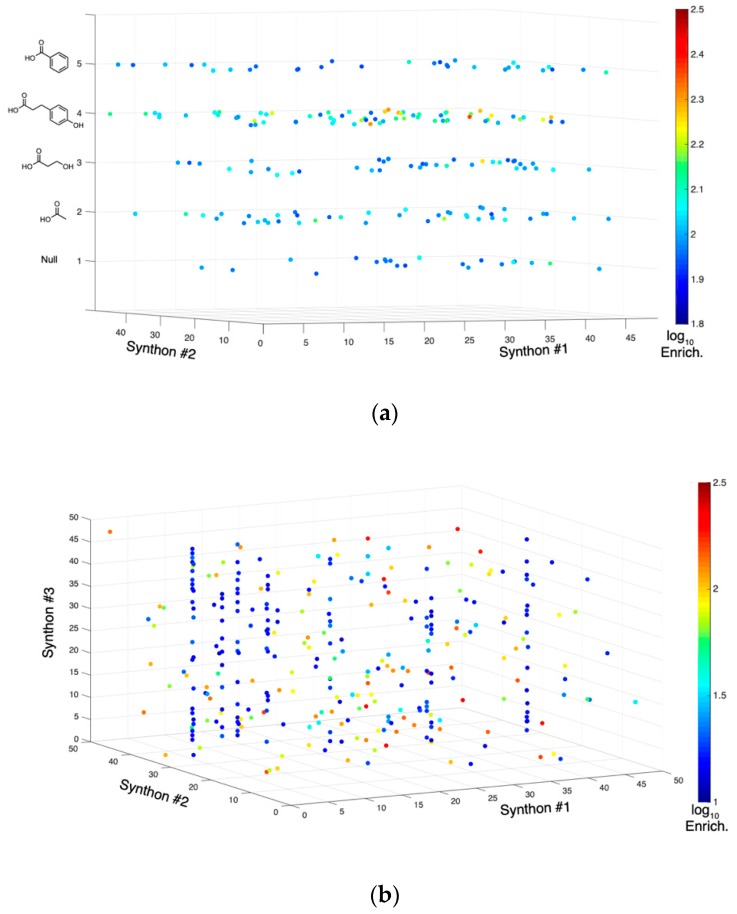
Cubic plot analysis of DNA-encoded chemical libraries (DEL) selection. (**a**) The highest 200 enriching members of the library encoded by the initial 48 codons (A library) are plotted indicating the synthon numbers of the first (*x*-axis), second (*y*-axis), and fourth (*z*-axis) chemical steps. (**b**) The highest 300 enriching members of the library encoded by the initial 48 codons (A library) that contained the phenol cap in the fourth step are plotted indicating the synthon numbers of the first (*x*-axis), second (*y*-axis), and third (*z*-axis) chemical steps. Color of the points corresponds to the log_10_ of the enrichment relative to the unselected library as indicated in the color bar scale.

The selection was then similarly applied to 9 fmol of the library (~10,000 molecules of each member) together with a LYNtide construct doped in at approximately 300,000 copies. In the library selection, the amount of enzyme used and incubation time was approximately ten times that required for full phosphorylation of a LYNtide-DNA conjugate. Selections were PCR amplified using sequencing adaptor and sample index containing primers. PCR amplicons were then sequenced and decoded. DNA sequencing analysis indicated 2100-fold enrichment of the LYNtide dope in, which was consistent with assay optimization experiments by qPCR. Within the library, several high enriching sequences were observed. A cubic plot of the top 200 enriching sequences based on their first, second, and forth synthon numbers (Figure 2a, Figure S4a) shows that the majority of these molecules contain the phenol cap in the fourth step, which is a positive indication that tyrosine kinase substrates were enriched. Plotting the first three synthon numbers of the molecules all containing the fourth step phenol cap (Figure 2b, Figure S4b) shows that the high enriching hits are quite distributed throughout the library, while several of the lower enriching hits (~10–100-fold) occur within lines, indicating these hits are among structurally similar families. In addition, none of the high enriching sequences were found in both of redundantly encoded libraries, which suggests randomness in the results likely due to under sampling. Recent analysis of DEL selection sequences has shown how under sampling can lead to low reproducibility and randomness in results [50]. Together, these results suggested the noise level in the selection was relatively high.

24 molecules were selected for follow up on the basis of both the presence of structural similar hits and overall enrichment (at least 10-fold) (Figure S5). To eliminate false positives, molecules were initially synthesized on a 20-mer DNA oligomer primer using identical procedures to those used in library construction. These crude products were placed on unique encoding DNAs by PCR. These constructs were then subjected to the substrate-based selection for hit validation using enrichment determined by qPCR (Figure S6). Of the molecules showing enrichment, six were selected for synthesis off encoding DNA scaffolds.

### 2.3. c-Src Activity and Binding Assays

Hit molecules were initially characterized as substrates for c-Src using the luciferase-based ADP-Glo assay (Promega). All six molecules appeared to be efficient substrates for Src kinase. We observed K_m_ values ranging from 3–6-fold higher than the LYNtide peptide substrate, with the lowest observed K_m_ being 13 µM for compound **SrcDEL14** (Table 1).

**Figure 3:**
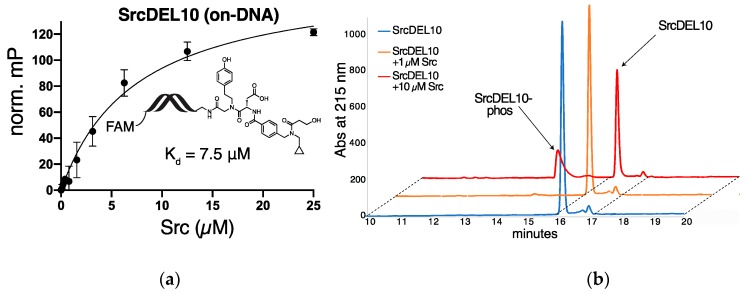
Validation of SrcDEL10 as a hit molecule from the substrate-mediated selection. (**a**) on-DNA binding affinity to c-Src measured by fluorescence polarization in the presence of ATP (100 µM). A **SrcDEL10** modified oligo was hybridized to a fluorescein amide (FAM) modified complementary oligo for the binding assay. (**b**) Off-DNA HPLC analysis of **SrcDEL10** (200 µM) with treatment of ATP (1 mM) and c-Src at high levels (1 µM, yellow, and 10 µM, red), which gave small amounts of the phosphorylated molecule. Peak identity was verified by LC/MS analysis (Figure S9).

Subsequently, we tested one of these hits, **SrcDEL10**, attached to a DNA scaffolds for binding affinity to Src kinase in the presence of ATP. These studies used the HPLC-purified ligand linked to 20-mer oligos, which were prepared for the prior qPCR validations under crude conditions. This construct was hybridized to a fluorescein-containing complementary oligo, and binding constants were determined by changes observed in fluorescence polarization, as previously described [51]. A binding constant of 7.5 µM was found for **SrcDEL10** in the presence of ATP (Figure 3a). **SrcDEL10** was selected for further study based on this result and the presence of the aspartic acid monomer, which is consistent with the typically acidic, natural peptide consensus sequences.

First, we sought to confirm the activity of **SrcDEL10** as a kinase substrate by LC/MS. Treatment of **SrcDEL10** with high levels of enzyme (1 µM) yielded very little (~1%) of the phosphorylated compound (Figure 3b). Under identical conditions, the LYNtide peptide was fully phosphorylated (Figure S7). This suggested that the signal observed in the ADP-Glo assay was likely due to ATP hydrolysis rather than phosphotransfer. To confirm this, compounds were tested with an NADH-based, coupled enzyme assay that also detects ADP generation [52]. For **SrcDEL5**, **8**, **10**, and **21**, ADP generation was observed in large molar excess over the compound, whereas ADP generation for LYNtide occurred at approximately one equivalent (Figure S8). This additionally supported that the ADP-Glo activity observed was due to activation of ATP hydrolysis and not phosphotransfer to these compounds. For **SrcDEL14** and **16**, little turnover was observed.

**Figure 4:**
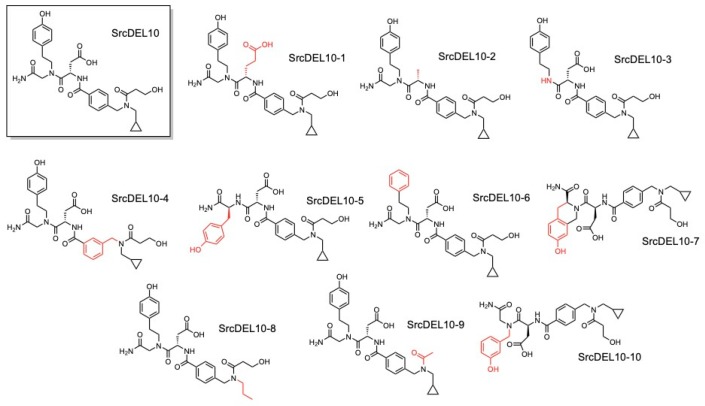
Synthesized derivatives of **SrcDEL10**.

**Figure 5:**
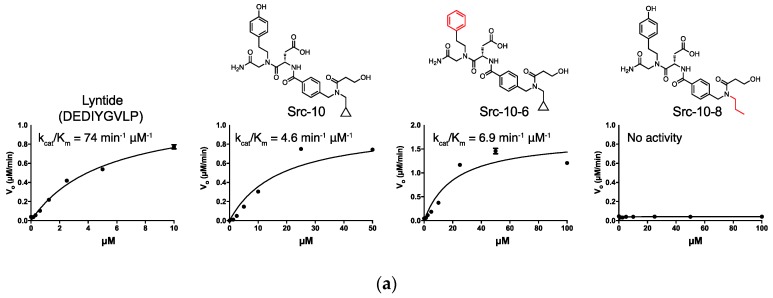

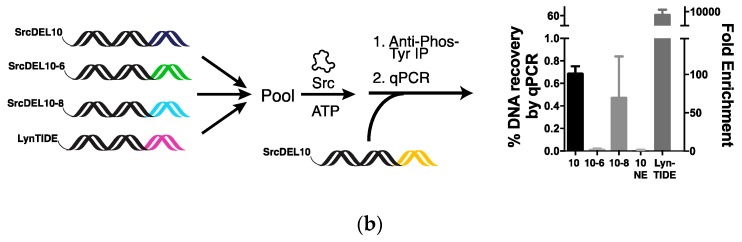
Structure activity relationship (SAR) studies of **SrcDEL10** derivatives. (**a**) ADP-Glo assays of the **SrcDEL10** derivative, **SrcDEL10**–**6**, lacking a phenolic oxygen show comparable activity to **SrcDEL10**. Derivative **SrcDEL10**–**8**, which contains the phenol but lacks the cyclopropyl group, shows no activity. (**b**) Analysis of enrichment in the substrate selection (shown in Scheme 1) shows the phenol is required for enrichment. (10-NE, **SrcDEL10** with no enzyme treatment).

We prepared 10 derivatives of **SrcDEL10** off-DNA to explore structure activity relationships (Figure 4). Initially, these were each assessed as substrates in the ADP-Glo assay (Table S3). Surprisingly, the assay indicated that **10**–**6**, which lacks a phenolic oxygen, was a comparable substrate to the parental compound (Figure 5a). Compound **10**–**8**, which lacks only the cyclopropyl group, lacked activity altogether. These results were potentially inconsistent with both the enrichment of **SrcDEL10** from the library by binding of the phosphorylated molecule to the 4G10 antibody and with the activity observed in the ADP-Glo assay being from a phosphotransfer to the small molecule.

We then questioned if **SrcDEL10** was indeed selected from the library based on the ability to serve as a Src substrate and accept a phosphate. Using purified DNA conjugates to **SrcDEL10**, **10**–**6**, and **10**–**8**, we confirmed that the phenolic oxygen was, in fact, required for enrichment in a qPCR selection assay and that the kinase treatment is also required for enrichment (Figure 5b). While the lower enrichment (100-fold) relative to LYNtide (10,000-fold) could be explained by differences in antibody affinity, the LC/MS assay would suggest this difference largely reflects the level of phosphorylation.

### 2.4. Src Activity Inhibition

**Figure 6:**
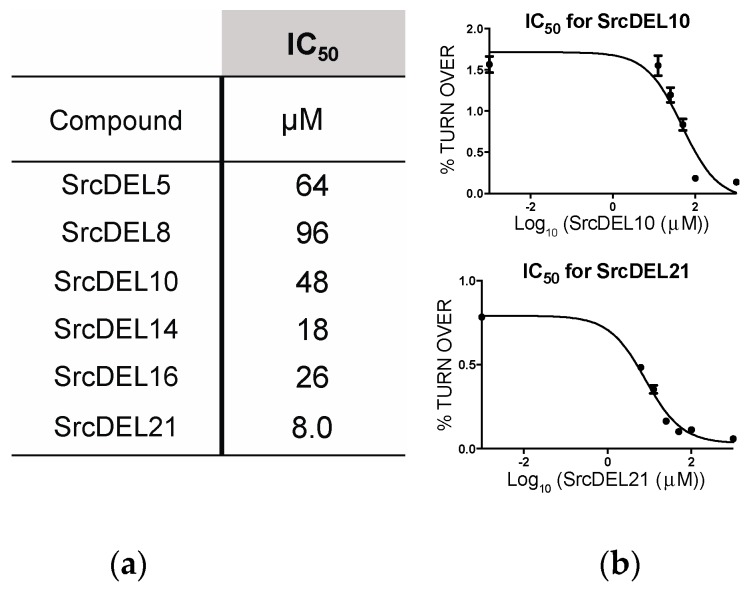
Inhibition of hit molecules in a radiolabel assay with ^32^P-ATP (50 µM) and biotinylated-LYNtide (50 µM) as substrates. (**a**) Half maximal inhibitory concentration (IC_50_) for six hit structures (structures shown in Table 1). (**b**) Representative data for **SrcDEL10** and **SrcDEL21**.

The original six off-DNA hits were tested for inhibition of c-Src activity. For these tests, we used ^32^P-ATP as a substrate and detected phosphorylation levels of a biotinylated-LYNtide substrate after adsorption to streptavidin coated membranes [16]. This assay should not be affected by activation of ATP hydrolysis or phosphotransfer to the small molecules. To avoid potential inhibition from reduction in ATP concentration, turnover levels were kept low (~1%), and ATP levels were confirmed to be negligibly reduced by ADP-Glo. Observed IC_50_ values ranged from 8.1 to 96 µM (Figure 6 and Figure S10). These values suggest modest binding affinity (~low µM) of the compounds given the use of 50 µM biotin-LYNtide (K_m_ = 5.2 µM) in these assays. Assay sensitivity was not sufficient for detection of such low turnover at significantly lower concentrations of biotin-LYNtide. For **SrcDEL10**, inhibition was additional validated by an HPLC assay of the LYNtide phosphorylation under similar conditions (Figure S11). There was no apparent correlation of these values to the K_m_ or k_cat_ values in the prior assays as substrates.

### 2.5. NMR Analysis

**Figure 7:**
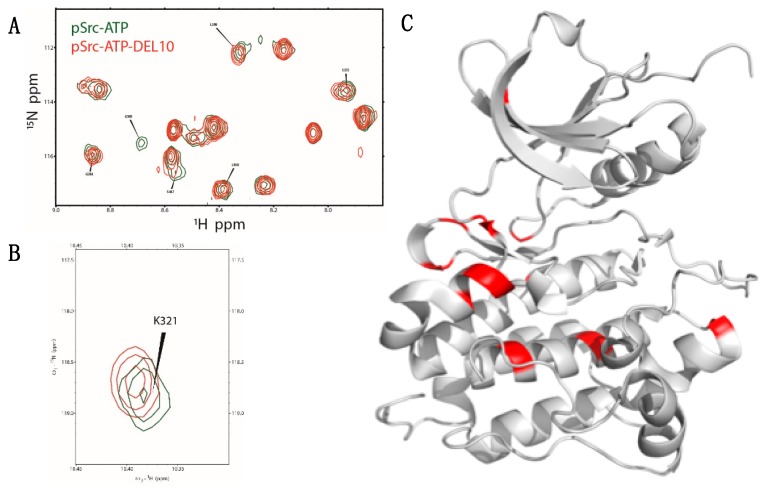
NMR analysis of **SrcDEL10** binding. (**a**) Overlay of ^15^N-HSQC spectra of the phosphorylated Src catalytic domain in the presence of ATP (green) or ATP and **SrcDEL10** (red). Ligand concentrations are near saturation based on estimated *K_m_* values. A portion of the spectrum showing significant changes is highlighted. See Figure SX for full spectrum. (**b**) A portion of the spectrum shows significant chemical shift perturbation of K321 in the presence of **SrcDEL10**. (**c**) Chemical shifts with large changes upon addition of **SrcDEL10** are mapped (red) to the ribbon drawing of the Src kinase domain (PDB ID: 1Y57) [58].

To gain insight in to the binding of **SrcDEL10** to c-Src, we performed NMR spectroscopy to measure changes in chemical shifts in the presence of **SrcDEL10**. Such changes are due to either the ligand altering the magnetic environment of protein amide groups through direct interactions, which can potentially identify specific residues that interact with the ligand, or to effects of binding on the overall protein conformational equilibrium. We collected ^1^H,^15^N heteronuclear single quantum coherence (^15^N-HSQC) spectra of the phosphorylated c-Src:ATP protein complex both in the presence and absence of **SrcDEL10**. Addition of the molecule produced chemical shift differences, or perturbations, as well as changes in peak intensity of resonances (Figure 7a,b, Figure S12). A number of c-Src:ATP resonances have low intensity, and some, such as G395, were lost or highly attenuated in the presence of **SrcDEL10**. Mapping the chemical shifts with changes to the Src crystal structure shows that the residues are mostly in the large lobe (bottom lobe as viewed in Figure 7c), but the changes are not sufficiently localized to one region to suggest a binding site. As protein kinases are well known to be conformationally flexible, it is not surprising that we observe changes in peak intensity and a broad spatial distribution of the affected residues, which likely reflects a more global conformational response to binding rather than direct interactions. Dispersed changes with ligand binding to kinases have been observed for many kinases, including protein kinase A, p38 kinase, FGFR1, and c-Src [53,54,55,56,57]. Our results suggest **SrcDEL10** affects c-Src conformational equilibrium, but the chemical shift perturbations are small and ligand association does not greatly alter the magnetic environment of c-Src amide groups at the binding site. Interestingly, binding was not observed in the absence of ATP.

### 2.6. Activity in Cell Culture

**Figure 8:**
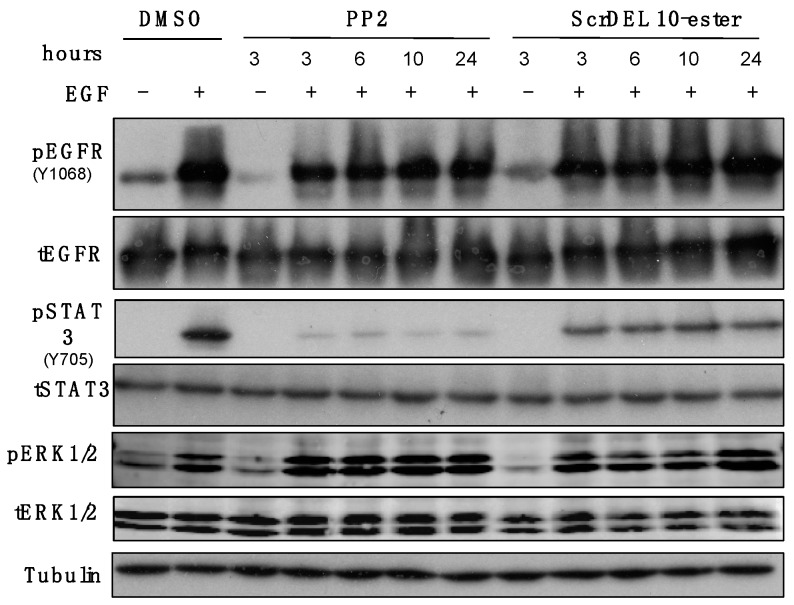
Inhibition of Src-dependent kinase signaling in EGFR-transformed mammary epithelial cells (NME) cells by **SrcDEL10**. NME cells were serum deprived for 24 h in the presence of vehicle control (DMSO), Src family inhibitor **PP2** (10 µM), or **SrcDEL10-ester** (100 µM) for the indicated amounts of time. Where indicated, these cells were subsequently stimulated with epidermal growth factor (EGF) (50 ng/mL) for an additional 30 min. Equal protein aliquots were analyzed by immunoblot with the indicated antibodies. Data are representative of at least 3 independent experiments.

We evaluated the ability of **SrcDEL10** to inhibit the activation of Src-dependent kinase signaling in EGFR-transformed mammary epithelial (NME) cells [59]. Epidermal growth factor (EGF) stimulation of NME cells leads to STAT3 phosphorylation via a Src-dependent process [60]. To improve the molecular properties for cell permeability, we prepared a methyl ester derivative of **SrcDEL10** at the aspartate side chain. Treatment of cells with this derivative at 100 µM prior to EGF stimulation showed a marked reduction in STAT3 phosphorylation levels (Figure 8). No reduction was observed with the parent **SrcDEL10** (data not shown). The broad specificity Src family inhibitor, **PP2**, was used as a positive control. Importantly, phosphorylation of ERK1/2, which occurs via a Src independent pathway, was not reduced. Similarly, autophosphorylation of EGFR at Y1068 was not affected. These results suggest that **SrcDEL10** is not a general protein tyrosine kinase inhibitor, but can specifically reduce Src-mediated signaling events.

## 3. Summary Discussion and Conclusion

We prepared a peptidomimetic DNA-encoded chemical library and applied a selection to enrich for substrates to the tyrosine kinase c-Src. Assessment of six hit molecules as substrates for c-Src in an assay that measures ADP production (ADP-Glo) gave K_m_ values in the mid-micromolar range (13–33 µM), which compares favorably to the K_m_ of an optimal peptide substrate LYNtide (K_m_ = 5 µM). Subsequent analysis indicated, however, that the observed activity was largely due to activation of ATP hydrolysis and not phosphotransfer for at least 4 of the 6 hits. Nonetheless, all six hit molecules displayed inhibitory activity in a radiolabel assay with IC_50_ values ranging from 8-100 µM. The ability to serve as a substrate was confirmed for a selected hit, **SrcDEL10**, by LC/MS. Application of the selection assay using DNA-linked **SrcDEL10** indicated that enrichment from the library was likely due to the ability to serve as a substrate, as enrichment was dependent on both the phenol oxygen and the enzyme treatment. Analysis of **SrcDEL10** with c-Src and ATP by NMR indicated binding, and an ester derivative demonstrated inhibition of Src signaling in NME cells.

The ATPase activity of protein kinases is well documented and is commonly observed background signal in many kinase assay approaches. Compared to phosphotransfer to an optimal peptide substrate, ATPase activity is generally dramatically lower (~two to three orders of magnitude) [61]. Here, the ATPase activity of c-Src (based on k_cat_/K_m_) was activated to within 10-fold of the LYNtide phosphotransfer activity. This is well above background levels of ATPase activity of Src alone, which were below the limit of detection under our assay conditions. Prior work has shown that ATPase activity can be regulated in parallel with protein kinase activity [62]. We hypothesize that **SrcDEL10** activates ATPase activity by binding to and stabilizing the active form of c-Src. The substrate and ATP hydrolysis activation capabilities may be connected, given activation was observed with several of the structurally diverse hits that were selected on the ability to accept a phosphate. Also, SAR of **SrcDEL10** derivatives indicated that the ATPase activation is likely a specific interaction. While this may be an uncommon phenomenon, it is a cautionary note as a potential source of assay interference for the study of kinase inhibitors and activators.

In our library selection, selection stringency was fairly low with a relatively high amount of enzyme used, which may be responsible for the modest ability of the hit **SrcDEL10** to serve as a true substrate. Setting the stringency of a selection appropriately may depend on the desired outcome of developing a substrate competitive inhibitor versus developing more active substrates. For inhibitors, there is no need to select for molecules that have high k_cat_ values. Based on prior work with c-Src, we presumed that it would be difficult for small molecules to serve as highly active substrates compared to larger (typically at least 9-mer) peptide substrates [24]. Further work may explore libraries which contain larger molecular weight molecules with increasing peptide character. For development of more active substrates, conducting multiple selections with titration of the enzyme down to low levels may be more appropriate. Also, combining both substrate and affinity selections may be a useful strategy. Part of the rationale for employing a substrate-mediated selection was the potential for sensitivity in enriching low affinity hits due to enzymatic turnover. High specific activity, which is a critical requirement for affinity-mediated selections, can be problematic for protein kinases. Our attempts at affinity-based selection with an immobilized c-Src were unsuccessful for either a DNA-linked LYNtide or **SrcDEL10**, which we presume was due to low specific activity of the enzyme as selections with ligands of low micromolar affinity are generally successful [63].

Given the applicability of substrate-mediated selections in both phage display and mRNA display [8,9], such selections should find additional application with DELs for both discovery of substrate competitive inhibitors and improved activity probes. Prior work from our lab has demonstrated DEL-compatible serine/threonine kinase, protease, and transferase substrate-mediated selections for activity detection using DNA-linked substrates [7]. We hope that this study will inspire others to apply these methods to DELs for discovery of new substrate molecules.

## 4. Materials and Methods

### 4.1. DNA Library Preparation

Following previously published methods [10], individual 40-mer oligonucleotides (Bioneer) containing 20 base common sequences and 20 bases of a unique variable region were treated with T4 polynucleotide kinase (PNK). Orthogonal 20 bases sequences for library assembly used were as previously reported [7,10]. Following, the 5′-phosphorylated oligos were combined based on complementarity in two pools so that Pool A contained variable regions 1–48 and Pool B contained variable regions 49–96 and then ligated by T4 DNA ligase. Following ligation, the pools were separately purified by PAGE, collected by electroelution, and then ethanol precipitated. DNA was quantified by UV absorbance and qPCR. Using approximately 10,000 copies of each library member as the template, each pool (variable sequences in each pool: Pool A = 1–48, Pool B = 49–96) was PCR amplified separately using with 0.5 μM end primers (one end primer contained the T7 promoter sequence), 1X DreamTaq buffer, 0.2 mM dNTPs, and 0.025 U/μL DreamTaq DNA polymerase for 20 cycles. The crude PCR products combined and by SPRI clean-up [64]. This was repeated using fresh aliquots of template until 1 nmol of each library was collected, as determined by UV and confirmed by qPCR using common end primers.

Each pool of dsDNA was transcribed 1X Thermo Scientific transcription buffer, 5 mM NTPs, 20 mM additional MgCl_2_, 200 nM dsDNA template (as determined by qPCR using T7-promoter primer), with 3 U/μL T7 RNA polymerase and incubated at 37 °C for 16 h. Upon incubation, a white precipitate was observed and 10 U DNase I was added and incubated at 37 °C for 30 min, followed by the addition of 1.5 eq. (relative to total Mg^2+^) of EDTA. RNA was purified using PureLink RNA Mini kits (Thermo Fisher) and quantified by UV absorbance.

Each pool of RNA at 12 μM (final concentration) was combined with 1X First Strand buffer (Invitrogen), 10 mM DTT, 1.0 mM dNTPs, 1.2 eq. (relative to RNA) of 5′-amine modified forward primer, and 1.0 U/μL SuperScript IV reverse polymerase. First, the RNA, dNTPs, and primer were incubated at 65 °C for 5 min and then placed on ice for at least 5 min. To this mixture, the buffer, DTT, and enzyme are added. The combined mixture was incubated at 47 °C for 16 h and purified as the RNA/DNA heteroduplex by SPRI clean-up. Total DNA was quantified by qPCR after single cycle PCR (primer extension).

### 4.2. Chemical Translation

Pools of A and B library RNA/DNA heteroduplex were hydrolyzed in 0.1 M NaCO_3_ buffer, pH 10.3 and heated to 95 °C for 5 min, then 1.5 eq. of all cognate common primers were added and the mixture was slowly cooled to RT. Following, the resulting ssDNA was taken up in 1X hybridization buffer (2X SSC, 10 mM Tris, pH 8, 1 mM EDTA, pH 8, 0.005% (*v*/*v*) Triton X-100, 0.02% (*w*/*v*) SDS, 0.5 mg/mL BSA, 0.5 mg/mL sheared salmon sperm DNA) to a final volume of 15 mL. Meanwhile, the array was pre-blocked with 1X hybridization buffer for 15 min at 37 °C, solution decanted and the ssDNA-containing hybridization solution was added to the array, and incubated at 37 °C for 36 h with agitation. Following hybridization, the solution was decanted from the array, and the array was washed 2 × 25 mL 1X hybridization buffer, 1 × 25 mL 10 × SSC, and 1 × 25 mL 0.25 × SSC. The array was then assembled into the transfer apparatus and hybridized DNA was eluted with 4 × 35 μL array elution buffer (10 mM NaOH, 1 mM EDTA, 0.005% Triton X-100), prepared fresh, and collected into a 384-well plate. Each elution was transferred to 20 μL DEAE Sepharose on a 384-well filter plate with 16 μL 60 mM HOAc and incubated for 5 min at RT. The second array elution was added to the DEAE-containing filter plate with an additional 16 μL 60 mM HOAc and incubated again for 5 min. The flow through was collected by centrifugation and transfer was repeated for the final two array elutions. The DNA-loaded filter plate was then washed 1 × 80 μL DEAE bind buffer, then 3 × 80 μL MeOH.

Building blocks added are shown in Table S1. Briefly, Fmoc-amino acids were coupled by adding 50 μL of 50 mM Fmoc amino acids, 50 mM EDC-HCl, 5 mM HOAt in 40% DMF/60% MeOH to the filter plate and incubated for 30 min at RT, similar to previous reports [38]. The reaction mixture was drained from the 384-well filter plate and a fresh reaction mixture was prepared as described above and incubated again for 30 min at RT. The filter plate was then washed 3 × 80 μL MeOH followed by 3 × 80 μL DMF. Following, 50 μL 20% (*v*/*v*) piperidine in DMF was added to each well following coupling of an Fmoc-amino acid and incubated for 30 min at RT. Each well was then washed 3 × 80 μL DMF followed by 3 × 80 μL MeOH. For peptoid monomers, chloroacetate was coupled by adding 50 μL of 100 mM sodium chloroacetate and 150 mM DMTMM-Cl in MeOH for 30 min at RT, with a repeated coupling using a fresh reaction mixture, as previously reported [39]. Following, resin was washed 3 × 80 μL MeOH and then 3 × 80 μL DMSO. For displacement, 50 μL of a 2 M stock of the desired primary amine in DMSO was added and incubated for 16 h at RT, followed by washing 6 × 80 μL DMSO, 3 × 80 μL MeOH, and 80 μL DEAE bind buffer. For benzyl-based peptoids, a solution of 100 mM 4-(chloromethyl)benzoic acid with 150 mM DIEA and 100 mM DMTMM-BF_4_ in DMF was prepared, added to the desired wells, and incubated at RT for 30 min. Coupling was repeated, and followed by washing with 3 × 80 μL DMF, 3 × 80 μL MeOH, 3 × 80 μL DMSO. Displacement was achieved followed the same procedure as with chloroacetate peptoids.

Prior to elution from DEAE Sepharose, all wells were washed 3 × 80 μL DMF, 3 × 80 μL MeOH, and lastly 80 μL DEAE bind buffer. For elution, 35 μL of DPCC DEAE elution buffer (10 mM NaOH, 1.5 M NaCl, 0.005% Triton X-100) was added and incubated for 5 min at RT. The elution was collected by centrifugation into a 384-well plate and elution was repeated for a total of four times. The collected elutions were pooled in a 50 mL 10 kDa MWCO centrifugal filter, neutralized with 1.0 eq. HOAc (relative to total NaOH) and Tris, pH 8.0 was added to a final concentration of 10 mM. The solution was then concentrated by centrifugation and washed 3 × TE. The recovery of translated library was quantified using qPCR.

Chemical translation was continued following the same steps as described by hybridization to Array C (step 1), Array A (step 2), and Array D (step 3), yielding the 3-mer DPCC library. Cartridge split for step 4. Following the completion of step 3, the 3-mer DPCC library was taken in 1X hybridization buffer (2X SSC, 10 mM Tris, pH 8, 1 mM EDTA, pH 8, 0.005% (*v*/*v*) Triton X-100, 0.02% (*w*/*v*) SDS, 0.5 mg/mL BSA, 0.5 mg/mL sheared salmon sperm DNA) to a final volume of 3.75 mL and then split across 5 cartridges (attached in series) containing variable sequences (denoted F5′-F9′) by incubation for 16 h at 37 °C with constant circulation by a peristaltic pump. The hybridization solution was collected and the cartridges were washed by 50 mL 1X hybridization buffer, 25 mL 10X SSC, and 25 mL 0.25X SSC. The cartridges were then removed and separately eluted with 2 mL array elution buffer and neutralized with HOAc.

Following quantification of the fully translated library by qPCR, the DNA was suspended in 1X DreamTaq buffer with 0.2 mM dNTPs, 1 μM reverse end primer, and 0.05 U/μL DreamTaq DNA polymerase for a single cycle PCR with a 20-min extension time to convert the translated ssDNA to dsDNA. The dsDNA was purified by SPRI clean-up. The translated dsDNA was deprotected by suspending in 250 mM sodium borate, pH 9.4 and then incubating for 2 h at 80 °C [65], followed by purification by SPRI cleanup and quantification by qPCR.

### 4.3. Substrate-Mediated Selection for Tyrosine Kinases

For optimization of substrate-based selection, two unique 180-mer constructs were prepared by PCR, as previously described [7]. Each contained two unique 20-mer sequences for specific amplification and detection in qPCR. One was prepared with an unmodified forward primer as a control. The other was prepared using a LYNtide 20-mer oligo conjugate as a primer.

For the selection of the library, 9 fmol of the deprotected translated dsDNA library was mixed with 500 zmol of a control LYNtide construct containing unique barcodes. This mixture was treated with recombinant c-Src kinase catalytic domain at approximately 2 μM concentration overnight at 30 °C. After kinase treatment, the mixture was phenol/chloroform extracted and precipitated from ethanol. 1.6 μL Protein G magnetic beads (ThermoFisher) were incubated with 10 μg anti-phosphotyrosine IgG. The antibody-loaded magnetic beads were washed with PBST with 1 mg/mL BSA, 1 mg/mL tRNAs) to remove any non-specifically bound antibody. Following, the precipitated kinase-treated library was taken up in 20 μL of the above buffer and incubated with the antibody-loaded magnetic beads. The magnetic beads were then washed 3 × 20 μL with the above buffer and 3 × 20 μL PBST. Captured library members were eluted by the addition of 20 μL of 1 mM phenylphosphate. The resulting supernatant was then used as a template in a PCR amplification with Illumina adaptor and indexes sequences, as previously described [7], and purified products submitted for analysis by Illumina MiSeq. Barcodes were assigned to codon numbers, as previously described [66], and enrichment calculated relative to the unselected library.

### 4.4. qPCR Assay

Kinase reaction was performed as follows 1x kinase buffer (25 mM Tris-HCl (pH 7.5), 2 mM dithiothreitol (DTT), 0.1 mM Na_3_VO_4_, 10 mM MgCl_2_), 1 mM ATP, different concentrations (1 μM to 100 μM) of substrates (either off DNA or on DNA hit molecules), and 12 nM rSrc by incubation for 2 h at 30 °C. After that, distinct 55mers were annealed to each kinase reaction, as previously described [67]. The annealed reactions were incubated with anti Pho-Tyr loaded protein G magnetic bead at RT for 30 min. After washing by three times 20 μL 1xPBST with 1 mg/mL BSA, 1 mg/mL tRNAs), the captured samples were eluted by 20 μL of 1 mM phenylphosphate. The resulting supernatant was then used as a template in qPCR.

### 4.5. ADP-Glo Assay

After the kinase reaction was performed as previous section, the reaction mixture (5 μL) was incubated with 5 µL of ADP-Glo™ reagent at RT for 40 min and then add 10 µL of kinase detection reagent at RT for 1 h. The luminescence was measure by plate reader.

### 4.6. NADH Coupled Assay

Kinase reaction was performed as follows 1x kinase buffer (25 mM Tris-HCl (pH 7.5), 2 mM dithiothreitol (DTT), 0.1 mM Na_3_VO_4_, 10 mM MgCl_2_), 1 mM ATP, 100 μM peptides and 12 nM Src at 30 °C for 3 h. The kinase reaction mixture was incubated with 30 unit/mL lactic dehydrogenase (LDH), 20 unit/mL pyruvate kinase (PK), 2 mM phosphoenolpyruvate (PEP), and 1 mM NADH. The absorbance of NADH was monitored at 340 nm.

### 4.7. Radiolabeled Assay

Each hit molecule was preincubated with 1.2 nM c-Src at 30 °C for 30 min. The kinase reaction was initiated with the addition of 50 μM LYNtide and 50 μM ATP with 0.15 μCi gamma ^32^P ATP at 30 °C for 10 min. The kinase reaction samples (2 µL) were spotted on P81 filter paper consisting of 1.5 cm squares. After air dry, the P81 papers were washed 4 times with 0.5% phosphoric acid for 5 min each. The P81 papers were washed one time with acetone for 5 min. The papers were then allowed to air dry for about 20 min. The dried P81 papers were analyzed by liquid scintillation counter.

### 4.8. Cell Signaling Assays

NME cells were constructed via stable overexpression of EGFR in normal murine mammary gland (NMuMG) cells as previously described [60]. These cells were cultured in DMEM containing 10% FBS, 10 μg/mL insulin, penicillin, and streptomycin. For cell signaling assays, cells were plated for 24 h prior to removal of the FBS for an additional 24 h. During this serum starvation, PP2 (Sigma, 529576) or SrcDEL10-ester were added as indicated, and cells were subsequently stimulated with EGF (50 ng/mL) (Goldbio, 1150-04) for 30 min. After this stimulation cells were lysed in a modified RIPA buffer containing 50 mM Tris, 150 mM NaCl, 0.25% Sodium Deoxycholate, 1.0% NP40, 0.1% SDS, protease inhibitor cocktail, 10 mM activated sodium ortho-vanadate, 40 mM β-glycerolphosphate, and 20mM sodium fluoride. Equal aliquots of these lysates were separated by reducing SDS PAGE and transferred to PVDF membranes. These membranes were probed with antibodies against phospho-EGFR Y1068, total EGFR, phosho-STAT3-Y705, total STAT3, phospho-ERK1/2, total ERK1/2 (Cell Signaling Technologies), and tubulin (DSHB). Differential binding of these antibodies was detected using their appropriate secondary antibodies.

### 4.9. Expression, Purification, and Phosphorylation of c-Src Catalytic Domain

The wild type c-Src catalytic domain (residues 255–533) was co-expressed with YoPH phosphatase in *E. coli* BL21 (DE3) cells and grow in M9 media containing ^15^NH_4_Cl. Uniformly labelled c-Src catalytic domain was purified by affinity and ion exchange chromatography as previously described [68]. To produce phosphorylated c-Src catalytic domain, purified protein was incubated in buffer containing 10-fold excess ATP, 20 mM MgCl_2_, 50 mM Tris-HCl (pH 8.0), 100 mM NaCl, 5% glycerol, and 1 mM DTT for 1.5 h at room temperature. ATP was washed out by exhaustive buffer exchange (20 mM Tris-HCl, pH 8.0, 100 mM NaCl, 5 mM DTT) for NMR samples.

### 4.10. NMR Experiments

NMR samples of ^15^N-labeled phosphorylated c-Src kinase domain were concentrated with a stirred concentrator. Final concentrations were phosphorylated [c-Src] = 200 µM, and [ATP] = 400 µM in the absence of SrcDEL10; and phosphorylated [c-Src] = 200 µM, [ATP] = 400 µM and [SrcDEL10] = 2 mM. The ^15^N TROSY-HSQC spectra were collected on an Avance Bruker 800 MHz spectrometer at 298K with 32 scans and 128 increments in the indirect ^15^N dimension.

## Figures and Tables

**Table 1:** Observed values for compounds in ADP-Glo assay.

	**K_m_**	**k_cat_**	**k_cat_/K_m_**	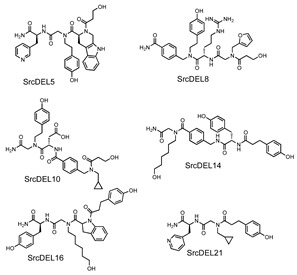
**Compound**	**(µM)**	**(min^−1^)**	**(min^−1^ µM^−1^)**	
SrcDEL5	33	350	11	
SrcDEL8	19	150	7.8	
SrcDEL10	18	82	4.6	
SrcDEL14	13	45	3.3	
SrcDEL16	18	32	1.8	
SrcDEL21	28	86	3.0	
LYNtide (DEDIYGVLP)	5.2	390	74	

## Supplementary Materials

The following are available online, Figure S1: Codon and gene abundances in the DNA-encoded library. Figure S2: Cubic plot analysis of a quality control DEL selection. Figure S3: Test enrichment assay with a LYNtide-DNA conjugate. Figure S4: Cubic plot analysis of DEL selection with B library. Figure S5: 24 hit molecules synthesized on-DNA for hit validation. Figure S6: qPCR assessed enrichment of initial 24 ligands on-DNA. Figure S7: HPLC analysis of LYNtide Phosphorylation. Figure S8: NADH coupled assay of hit molecules with c-Src. Figure S9: LC/MS analysis of **SrcDEL10** phosphorylation. Figure S10: IC_50_ curves for selected hits in the ^32^-P-ATP inhibition assay. Figure S11: HPLC-assessed inhibition of LYNtide phosphorylation by **SrcDEL10**. Figure S12: Full HSQC NMR spectrum for **SrcDEL10** binding to c-Src:ATP. Table S1: Library monomer set for the first 3 synthetic cycles. Table S2: Library monomer set for synthetic cycle 4. Table S3: ADP-glo assay of **SrcDEL10** derivatives. Supplementary methods and compound characterization.
