# Supplementary material for: Application of a Substrate-Mediated Selection with c-Src Tyrosine Kinase to a DNA-Encoded Chemical Library
Source: Molecules. 2019 Jul 30;24(15):2764. doi: 10.3390/molecules24152764 (PMC6695731; doi:10.3390/molecules24152764)
Supplement: Supplementary file 1 [file molecules-24-02764-s001.pdf]

Supplementary Information

Application of a Substrate-mediated Selection with c-Src Tyrosine Kinase to a DNA-Encoded Chemical Library

Dongwook Kim [1, 2, #], Yixing Sun [1, #], Dan Xie [1], Kyle E. Denton [1, 3], Hao Chen [1], Hang Lin [1], Michael K. Wendt [1], Carol B. Post [1], and Casey J. Krusemark [1,*]

[1] Department of Medicinal Chemistry and Molecular Pharmacology, Purdue University Center for Cancer Research, Purdue University, West Lafayette, IN 47907, USA; sun646@purdue.edu (Y.S.); xie243@purdue.edu (D.X.); chen2334@purdue.edu (H.C.); lin79@purdue.edu (H.L.); mwendt@purdue.edu (M.K.W.); cbp@purdue.edu (C.B.P.)

[2] Present address: Dencoda, LLC, West Lafayette, IN 47906, USA; dwkim@hotmail.com (D.K.)

[3] X-Chem Pharmaceuticals, Waltham, MA 02435, USA; kdenton@x-chemrx.com (K.E.D.)

# These authors contributed equally to this work.

* Correspondence: cjk@purdue.edu (C.J.K.)

Figures

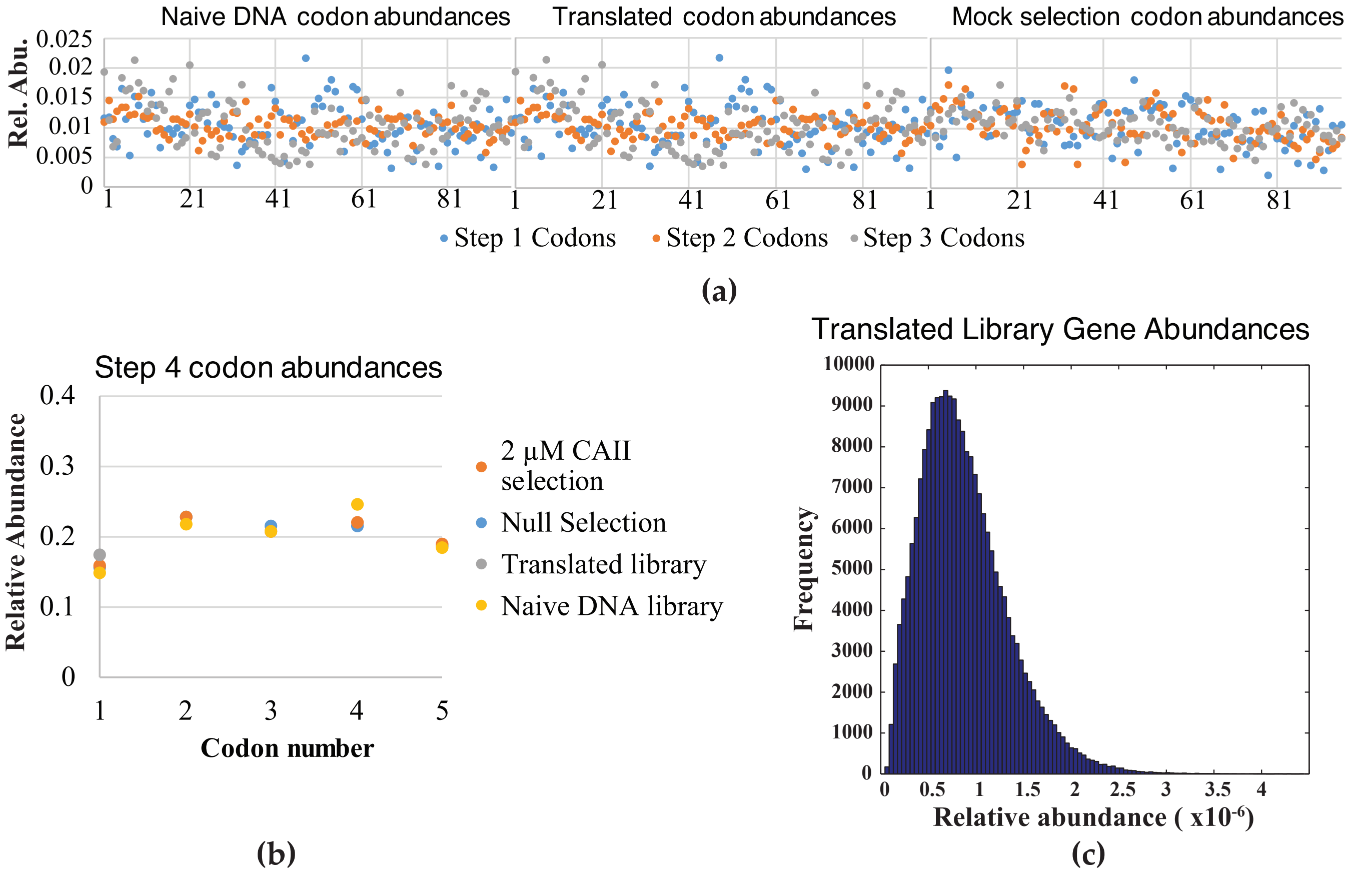

Figure S1. Codon and gene abundances in the DNA-encoded library. (**a**) Observed relative codon abundances for codons encoding chemical steps 1-3 of the naïve DNA prior to library generation, the chemically translated library, and the library after a mock selection (biotin-blocked streptavidin beads). (**b**) Observed relative codon abundances for codons encoding chemical step 4. (**c**) Histogram of the relative gene abundances of the 2.2 x 10^5 genes encoding chemical steps 1-3 only in the translated, unselected library. Gene abundances were approximated by the product of the individual codon abundances.

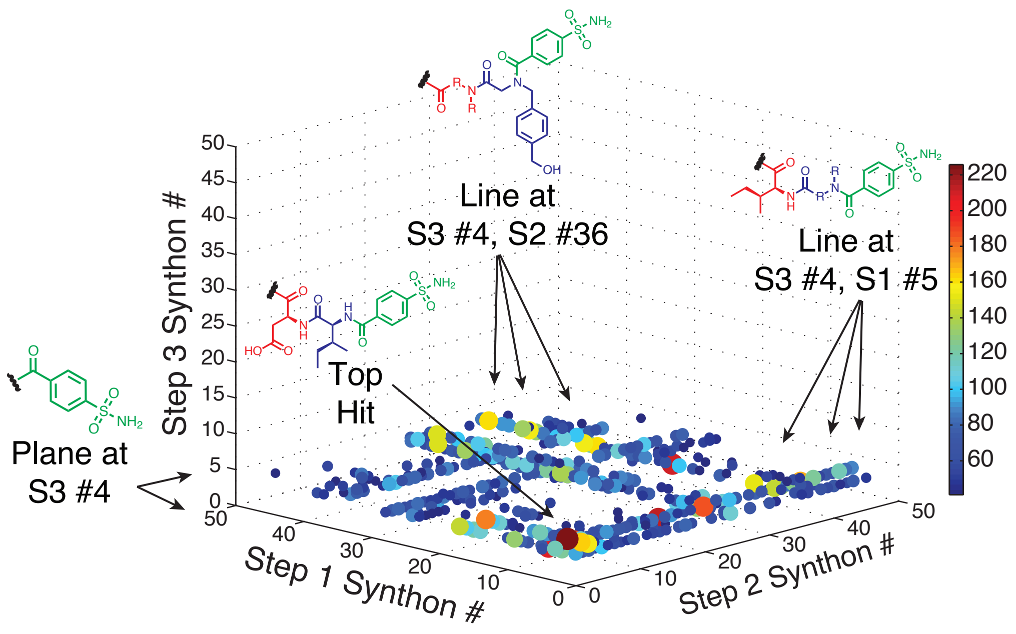

Figure S2. Cubic plot analysis of a quality control selection against bovine carbonic anhydrase II (CAII). The highest 400 enriching members of the library encoded by the initial 48 codons (A library) are plotted indicating the synthon numbers of the first (y-axis), second (x-axis), and third (z-axis) chemical steps. Note all 400 top enriching members are within the plane for synthon 4 at chemical step 3, which contains the known CAII pharmacophore, an aryl sulfonamide. The library was selected after 3 steps of chemistry against immobilized CAII at an approximate concentration of 2 μM. Dot size and color (according to the heat scale at right) represent fold enrichment relative to the unselected library.

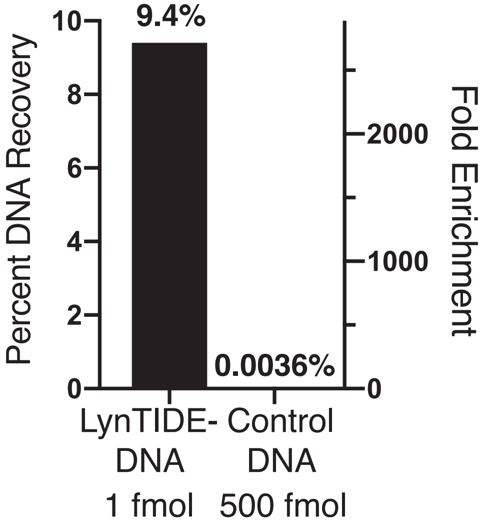

Figure S3. Test enrichment assay with a LYNtide-DNA conjugate. In a model enrichment assay, enrichment for tyrosine kinase substrates was detected by qPCR of model 180-mer constructs. Full assay method description is provided in *Methods and Materials 4.4 qPCR Assay*.

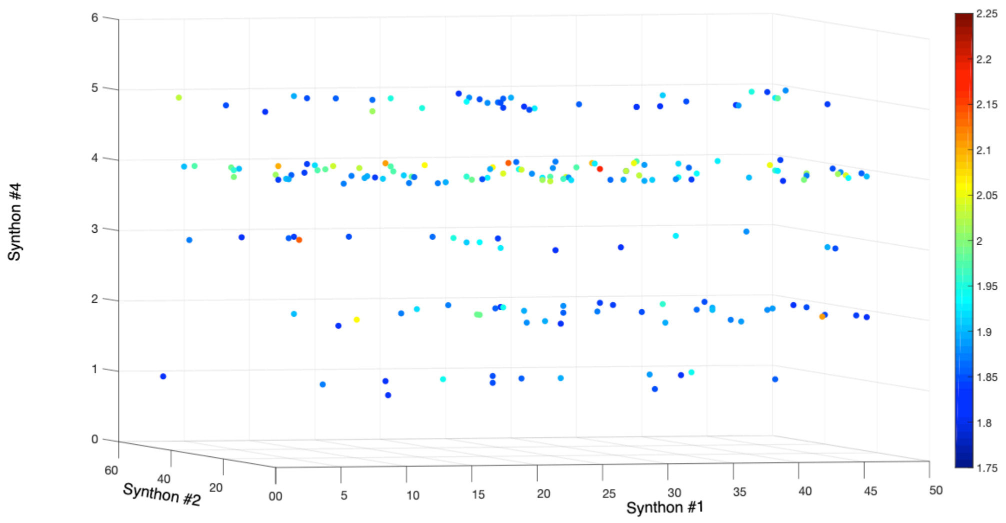

(**a**)

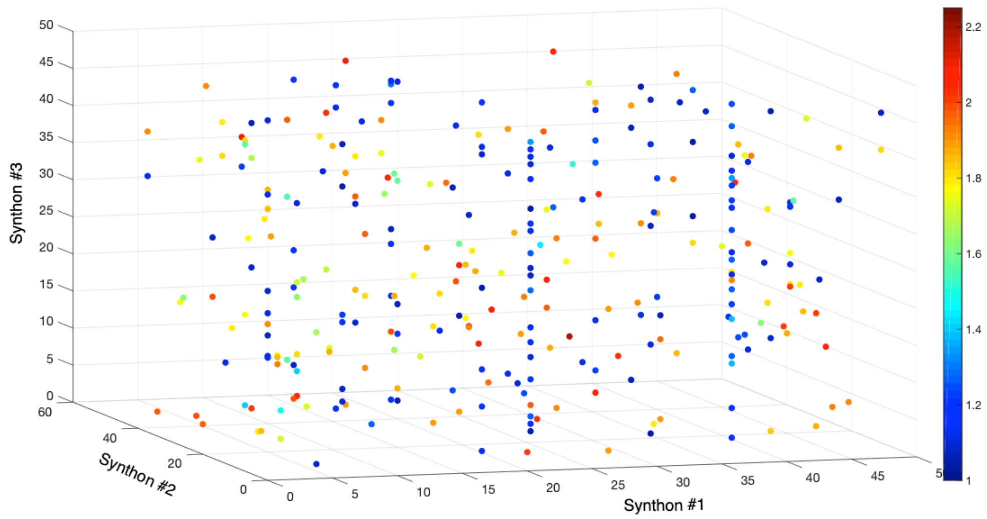

(**b**)

Figure S4. Cubic plot analysis of DEL selection with B library, as in main text Figure 2. (**a**) The highest 200 enriching members of the library encoded by the initial 48 codons (B library) are plotted indicating the synthon numbers of the first (x-axis), second (y-axis), and fourth (z-axis) chemical steps. (**b**) The highest 300 enriching members of the library encoded by the initial 48 codons (B library) that contained the phenol cap in the fourth step are plotted indicating the synthon numbers of the first (x-axis), second (y-axis), and third (z-axis) chemical steps. Color of the points corresponds to the $\log_{10}$ of the enrichment relative to the unselected library as indicated in the color bar scale.

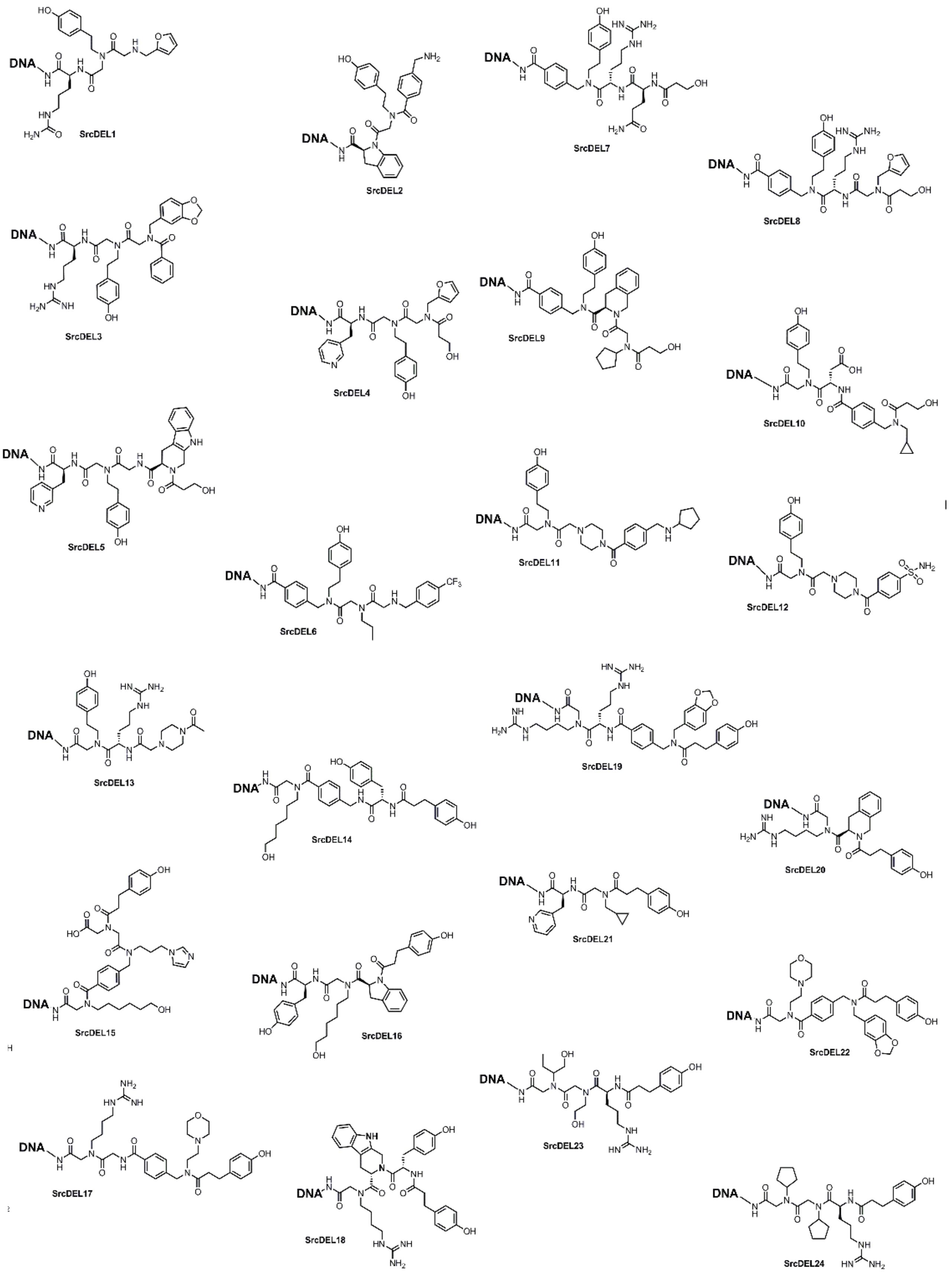

Figure S5. 24 hit molecules synthesized on-DNA for hit validation.

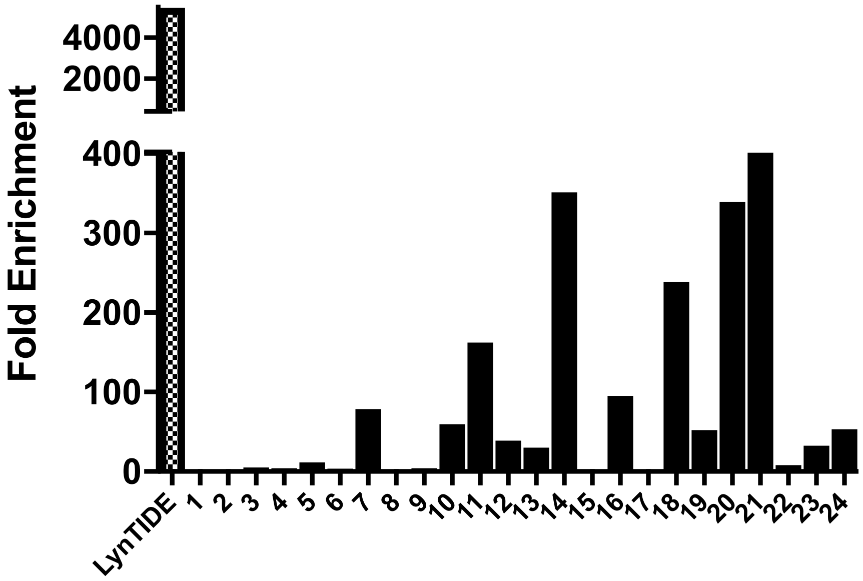

Figure S6. qPCR assessed enrichment of initial 24 ligands on-DNA. Enrichment was calculated relative to a non-ligand control. Assays were conducted as described in *Methods and Materials 4.4 qPCR assay.*

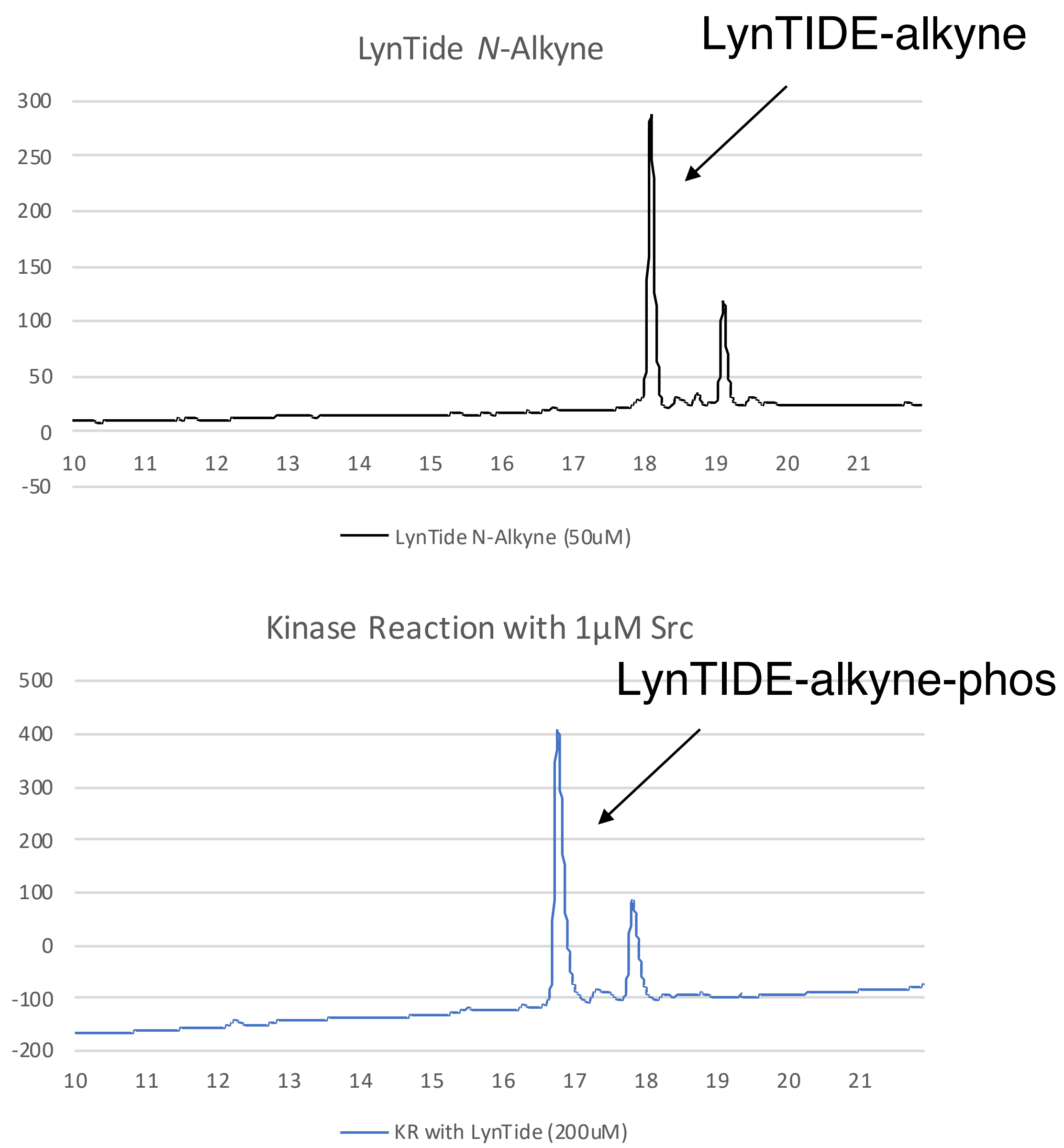

Figure S7. HPLC Analysis of LYNtide Phosphorylation. The kinase reaction was performed by incubating 200 μM LYNtide with Src in 1x kinase buffer (as outlined in section *4.6 NADH coupled assay*, with 1mM ATP) for 30 min. Treatment with low levels (1 μM) of c-Src gave full phosphorylation. Peak identities were verified by LC/MS. The peak at 19 minutes is an impurity in the LYNtide alkyne preparation, which was also phosphorylated.

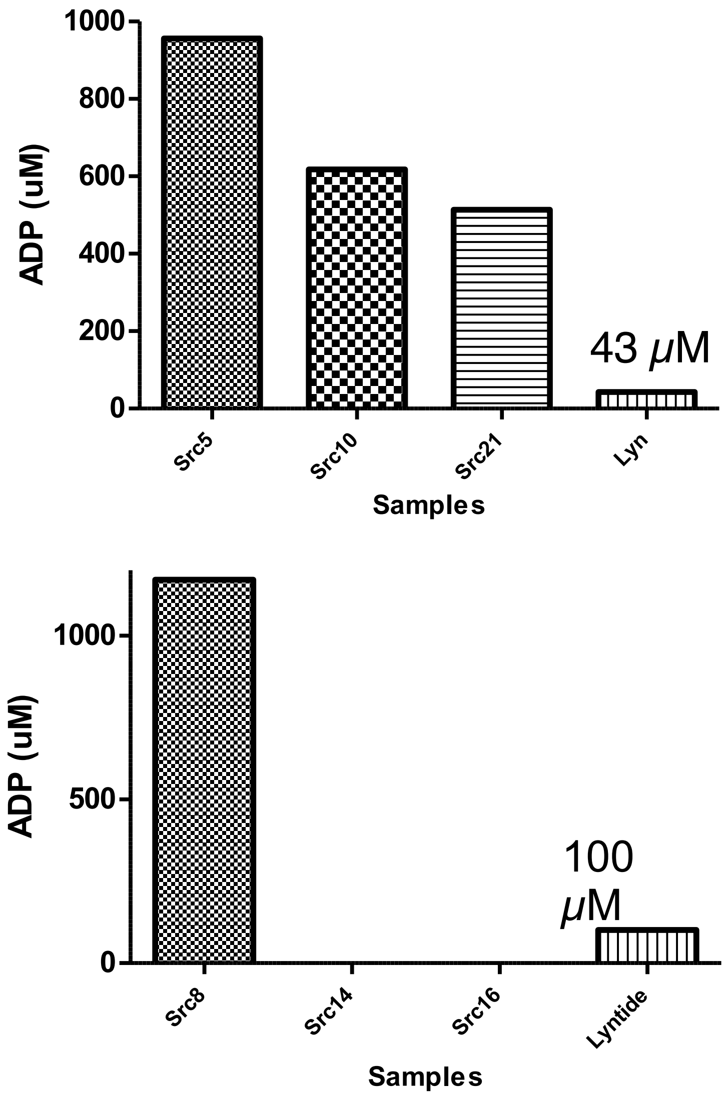

Figure S8. NADH Coupled Assay of Hit Molecules with c-Src. Samples were composed of 1 mM ATP, 100 μM putative substrate molecules, and 12 nM c-Src kinase at 30 C for 3 hours. Full method description is provided in *Methods and Materials 4.6 NADH assay.*

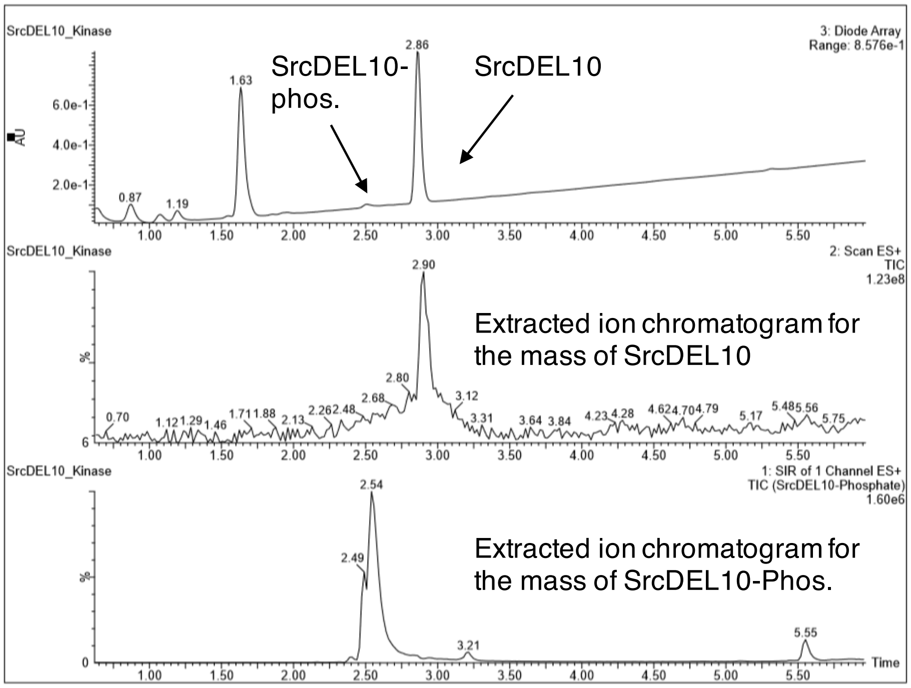

(**a**)

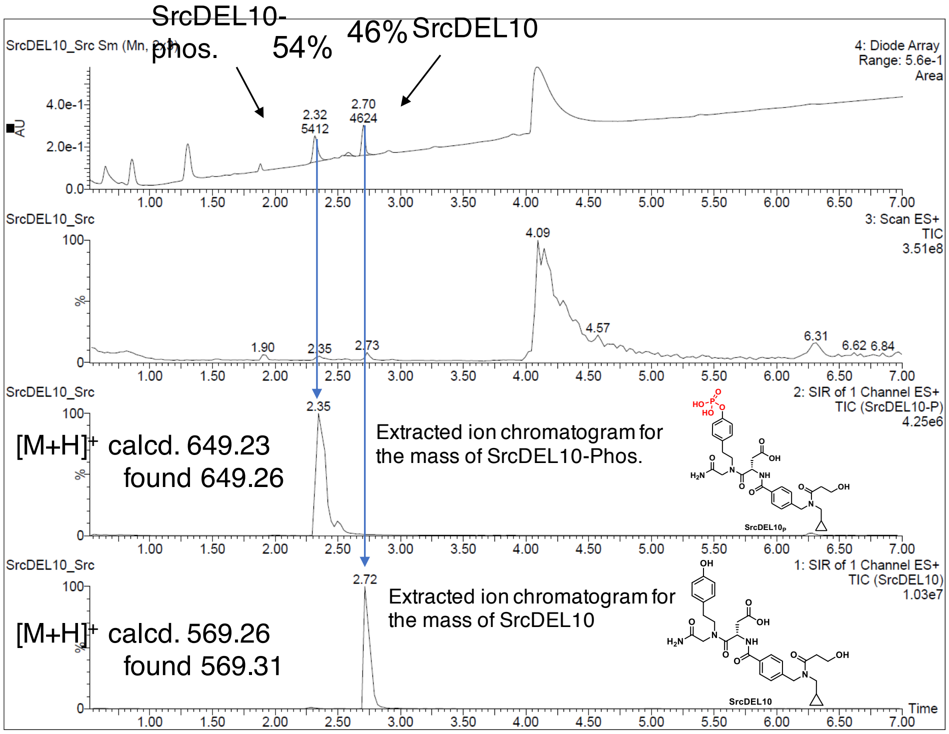

(**b**)

Figure S9. LC/MS analysis of **SrcDEL10** Phosphorylation. (a) **SrcDEL10** (100 μM) was treated with 100 μM ATP and 0.3 μM c-Src at 30 C for 16 hours. (b) **SrcDEL10** (100 μM) was treated with 100 μM ATP and 10 μM c-Src at 30 C for 16 hours.

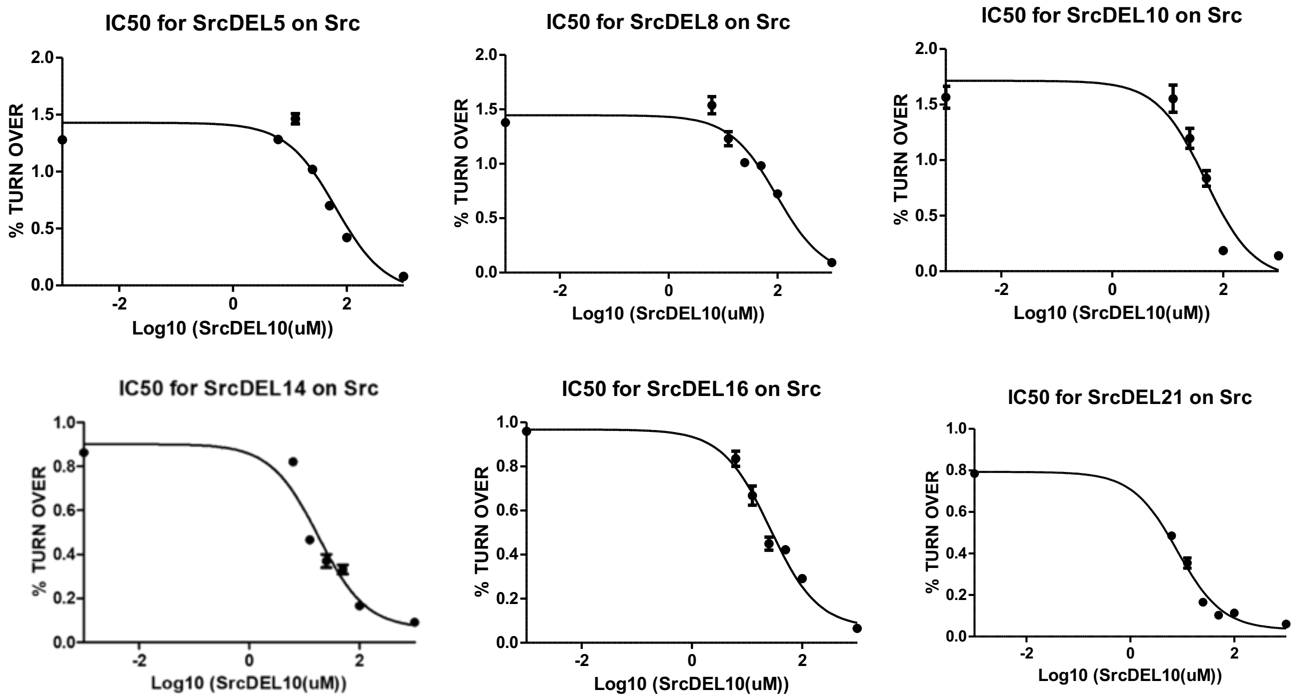

Figure S10. IC_{50} Curves for all compounds in the ^{32}P-ATP inhibition assay. Assay methods are described in *Methods and Materials.*

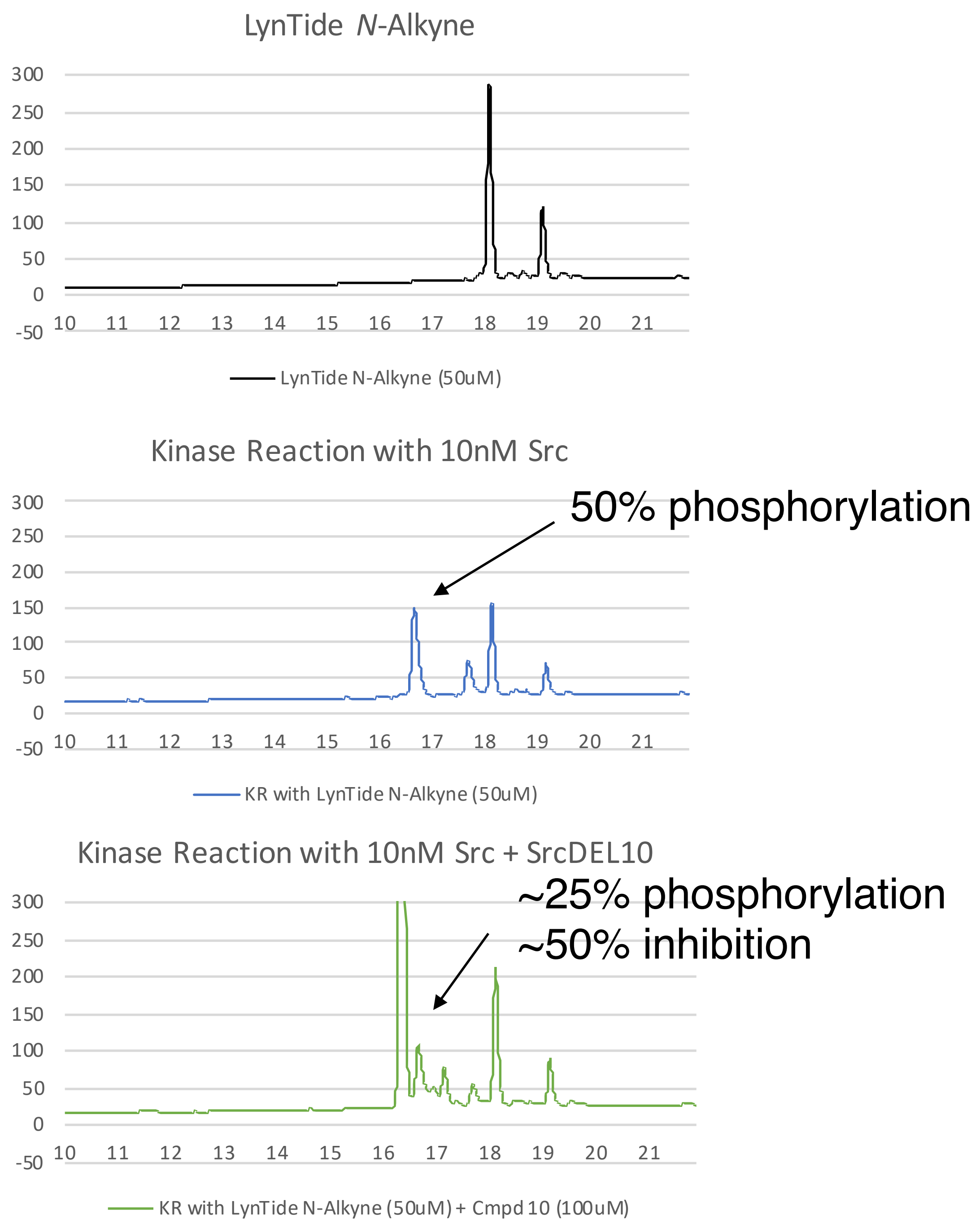

Figure S11. HPLC-assessed Inhibition of LYNtide Phosphorylation by **SrcDEL10**. The kinase reaction was performed by incubating 50 µM LYNtide with 10 nM Src (preincubated with 100 µM **SrcDEL10** for 30 min, if applicable) in 1x kinase buffer (as outlined in *4.6 NADH coupled assay,* with 1mM ATP) for 30 min.

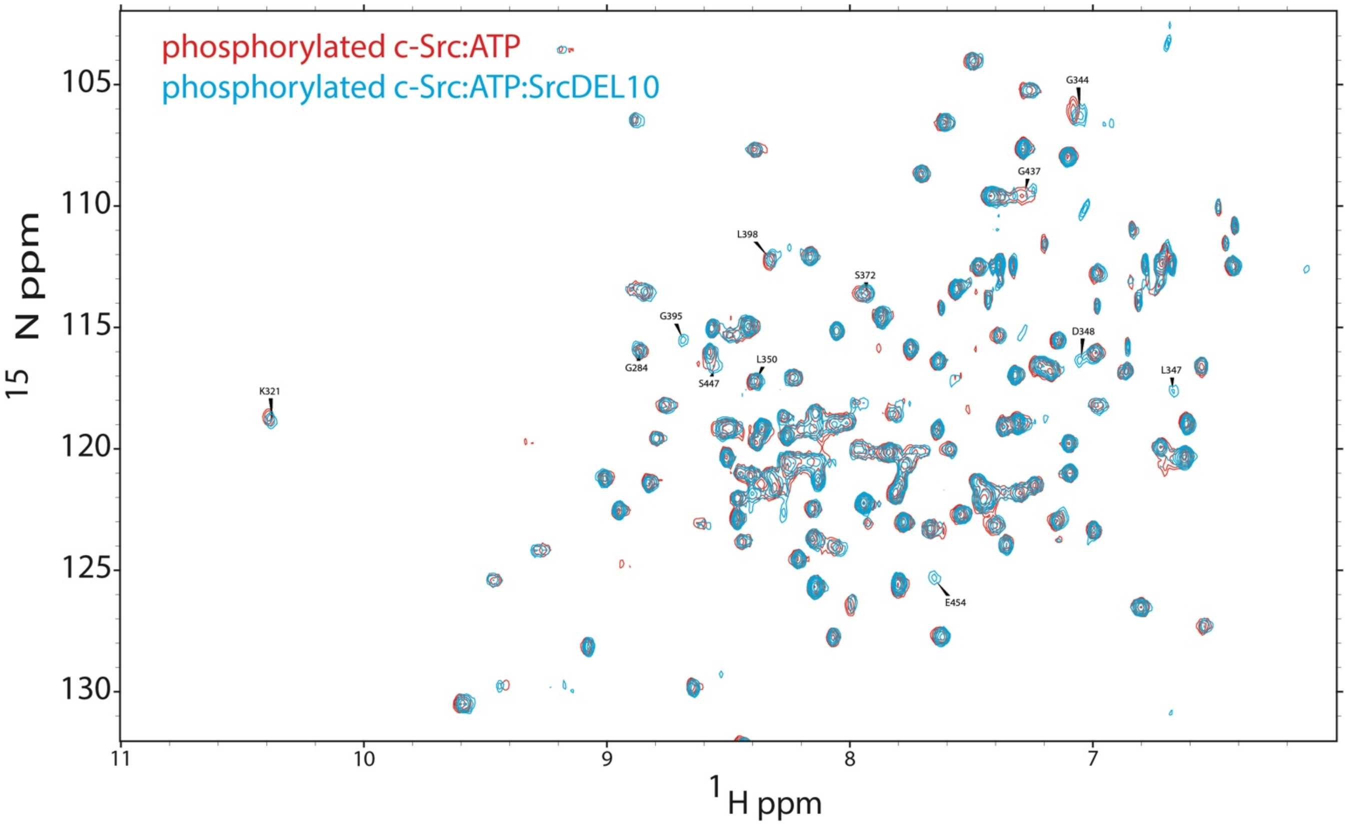

Figure S12. Full HSQC NMR Spectrum for **SrcDEL10** binding to c-Src:ATP.

Table S1a. Library monomer set for first 3 synthetic cycles.

Synthon #	Structure	Coupling Yield	Synthon #	Structure	Yield (SN2, Cl-Ac)
1	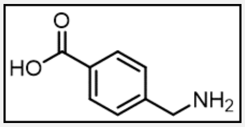	>95%	25	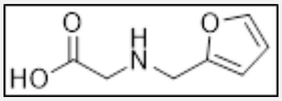	>95%, >95%
2	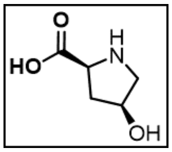	>95%	26	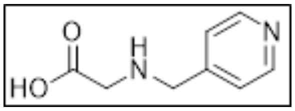	>95%, 50%
3	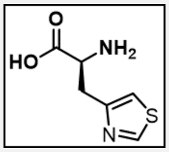	90%	27	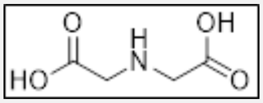	>95%, 95%
4	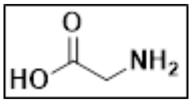	ND[1]	28	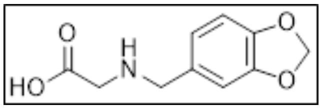	>95%, >95%
5	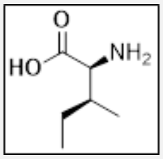	ND[1]	29	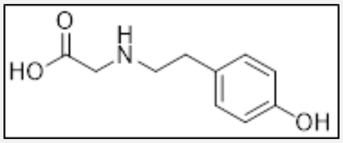	>95%, 90%
6	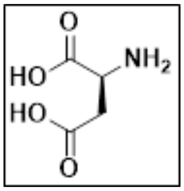	ND[1]	30	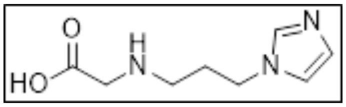	>95%, >95%
7	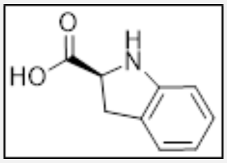	ND[1]	31	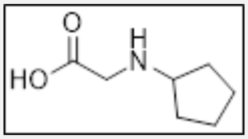	>95%, >95%
8	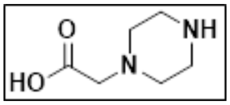	>95%	32	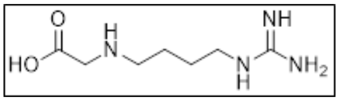	>90%, 60%

9	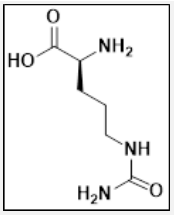	>95%	33	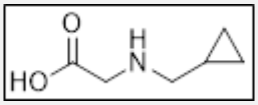	>95%, >95%
10	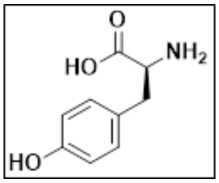	>95%	34	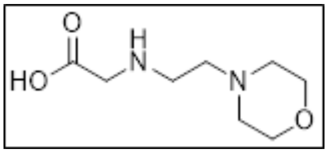	>95%, >95%
11	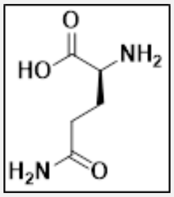	85%	35	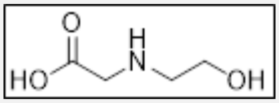	>95%, >95%
12	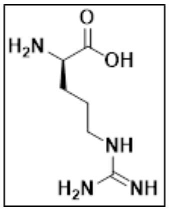	>95%	36	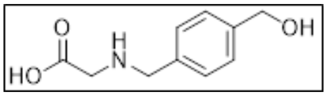	>80%, ND
13	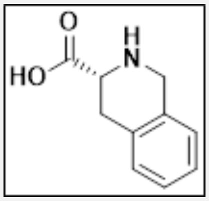	>95%	37	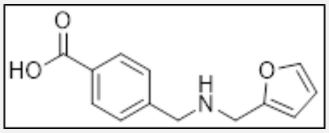	ND[2]
14	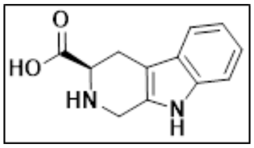	>95%	38	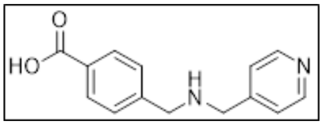	ND[2]
15	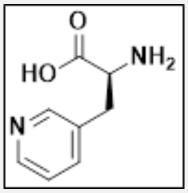	90%	39	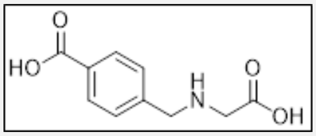	ND[2]
16	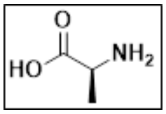	ND[1]	40	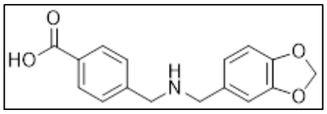	ND[2]
17	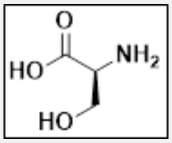	ND[1]	41	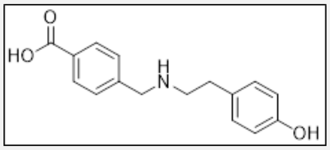	ND[2]

18	N/A	N/A	42	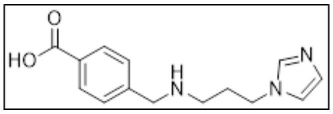	ND[2]
19	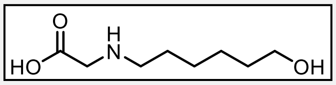	>95%, >95%	43	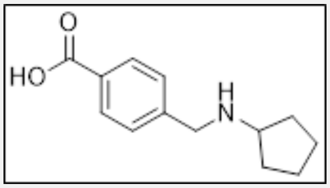	ND[2]
20	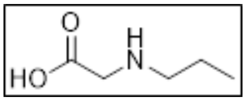	>95%, >95%	44	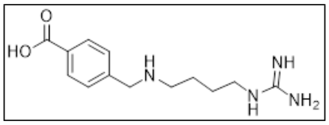	ND[2]
21	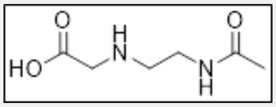	>95%, ND	45	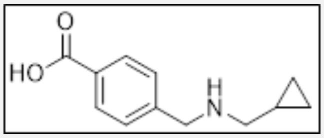	ND[2]
22	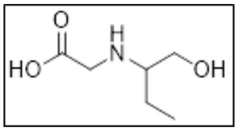	>95%, >95%	46	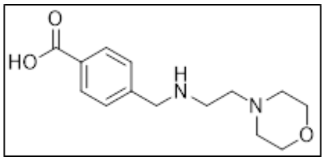	ND[2]
23	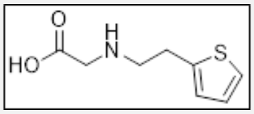	>95%, >95%	47	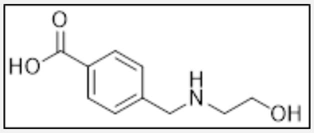	ND[2]
24	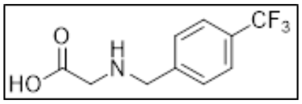	>95%, >95%	48	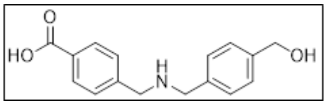	ND[2]

Notes: ND: Not determined. [1] Efficient on-DNA chemistry was previously reported [1]. [2] These primary amines were tested only using the standard peptoid chemistry (Synthons 25 to 36).

Table S1b. Building block exceptions at Synthon #3. (For QC purposes only.)

Name	Synthon #	Structure	Name	Synthon #	Structure
CBS	4	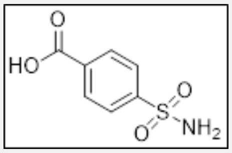	AcNT2AA	20	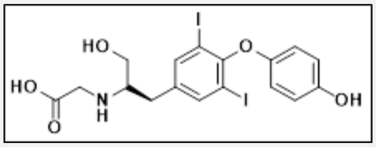

Table S2. Library monomer set for synthetic cycle 4.

Name	Building Block #	Structure
Null	1	N/A
Ac	2	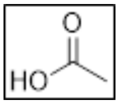
SerOH	3	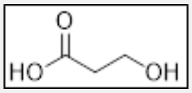
TyrOH	4	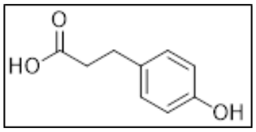
Bz	5	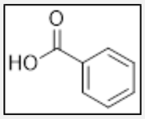

Table S3. ADP-Glo assay of **SrcDEL10** derivatives.

Enzyme	Substrate	Km (uM)	Vmax (uM/min)	Kcat (1/min)	Kcat/Km (1/min/uM)
Src	Src10	17.67	1.75	145.7	7.77
	Src10-1	20.69	1.11	92.84	4.49
	Src10-2	4.31	0.492	41.02	9.52
	Src10-3	3.79	0.382	24.21	6.39
	Src10-4	9.68	0.54	44.66	4.61
	Src10-5	4.37	1.078	89.33	20.56
	Src10-6	21.13	1.738	144.9	6.86
	Src10-7	15.45	1.47	122.8	7.95
	Src10-8	>100	N	N	N
	Src10-9	>100	N	N	N
	Src10-10	>100	N	N	N
	Lyntide	5.23	1.165	388.3	74.24

Supplementary Methods and Compound Characterization

Control Selection Against Bovine Carbonic Anhydrase II

Bovine carbonic anhydrase (Sigma) was chemically biotinylated similar to prior work [2]. Briefly, to 40 μL of 220 μM solution of bovine carbonic anhydrase (B. CAII) in PBST, pH 7.4, 5 μL of 52 mM NHS-LC-Biotin (Thermo-Fisher) in DMSO was added and incubated at RT for 1.5 hours. The mixture was diluted with PBST and concentrated using a 10 kDa MWCO centrifugal filer (10,000 x g) and then washed and re-concentrated 5x. The concentration of biotinylated B. CAII was determined by UV absorbance. Nanolink Streptavidin magnetic beads (11 μL) were washed 3x 11 μL PBST (0.1 M sodium phosphate, 0.15 M NaCl, 0.02% Tween-20) with 1 mg/mL BSA and 1 mg/mL tRNAs. Biotinylated B. CAII (1.2 eq. based on magnetic bead capacity) in the above buffer was incubated with the pre-washed magnetic beads at RT for 1 hour, followed by the addition 100 eq. biotin (final 5% DMSO) for 30 minutes. The resulting solution was removed and the magnetic beads were then washed 3x 20 μL PBST with 1 mg/mL BSA and 1 mg/mL tRNAs to remove any non-immobilized B. CAII. The CAII-loaded magnetic beads were then split into two portions: one with 10 μL beads (for a ~20 μM protein selection) and another with 1 μL beads for a 2 μM protein selection. Separately, 10 μL of Nanolink Streptavidin magnetic beads were pre-washed and blocked with biotin for a null selection. The previously deprotected translated dsDNA library (500 fmol) was suspended in the above buffer (final volume of 30 μL) and split between the two CAII loaded magnetic beads and null (10 μL each) and incubated at RT for 1 hour. Following, the solution was removed and washed with the above buffer 5x 10 μL. Elution from the magnetic beads was completed by suspending the beads in 10 μL H_2O and heating at 95 °C for 5 minutes. The resulting supernatant was then submitted for PCR amplification and analysis by next generation DNA sequencing.

Synthesis of DNA-5′-LYNtide

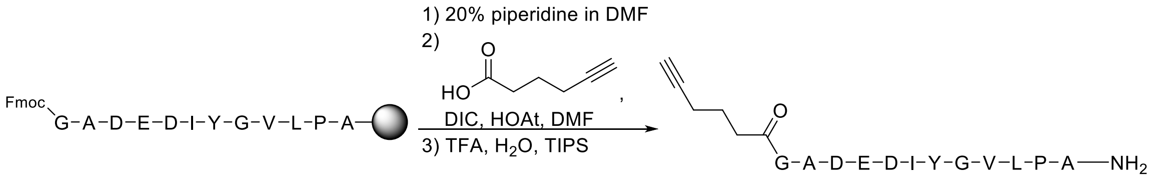

Solid Phase Synthesis of LYNtide Alkyne. LYNtide-loaded resin was purchased from GenScript. Resin was swelled in DCM and then 20% piperidine in DMF was applied to deprotect the N-terminal Fmoc group. The deprotection was performed by a double 20-minute incubation at RT. A mixture containing 5-hexynoic acid (3 eq.), DIC (3 eq.), and HOAt (3 eq.) in DMF (1 mL) was then applied. The amide bond formation was performed by a double 2-hour coupling at RT. Peptide was cleaved off the resin by incubating in a cleavage cocktail (95:2.5:2.5 TFA:H_2O:TIPS) for 4 hours followed by a precipitation in cold ether. The resulting precipitate was subsequently purified by semi-prep HPLC at a flow rate of 5 mL/min with H_2O (0.1% TFA) and MeCN (0.1% TFA) as mobile phase. The peptide was characterized by ESI/MS. m/z: $[M+H]^+$ calcd. for $C_{60}H_{90}N_{13}O_{20}^+$, 1312.6, found $[M+H]^+$, 1312.7, $[M+2H]^{2+}$, 657.2.

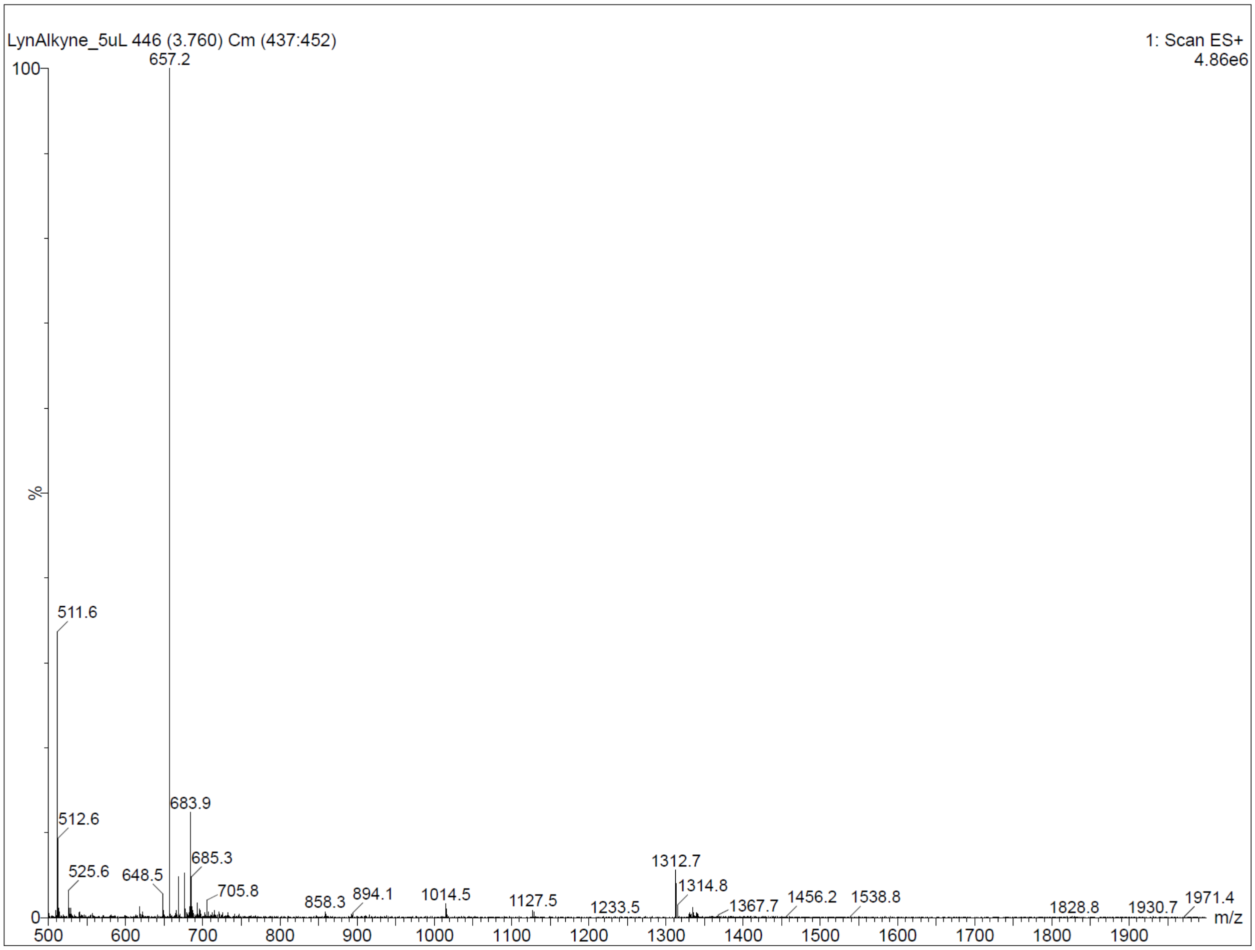

Synthesis of 6-azidocaproic acid (Cap(N_3)) was conducted as previously described.[1]

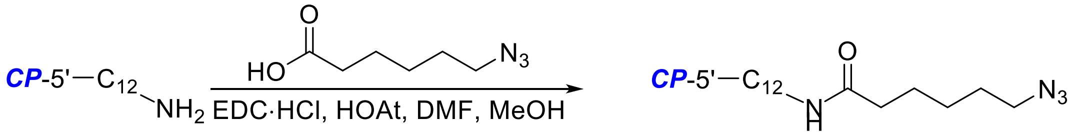

Synthesis of DNA-5′-Cap(N_3). Amine modified 20-mer common primer (CP) oligo (CP-C_{12}-NH_2, synthesized by Integrated DNA technologies) was immobilized on DEAE Sepharose with DEAE bind buffer and then equilibrated with MeOH. A mixture containing 6-azidocaproic acid (50 μM) (prepared as previously described [3]), EDC·HCl (50 μM), and HOAt (5 μM) in 40:60 DMF:MeOH was applied to and incubated with the solid phase. The amide bond formation was performed by a double 20-minute coupling at RT. The modified oligo was eluted with 1 mL of DEAE elution buffer and purified by semi-prep HPLC at a flow rate of 1 mL/min with H_2O (0.75% HFIP, 0.75%TEA, 5μM EDTA, pH 7.0) and 10:90 H_2O:MeOH (0.75% HFIP, 0.75%TEA, 5μM EDTA, pH 7.0) as mobile phase.

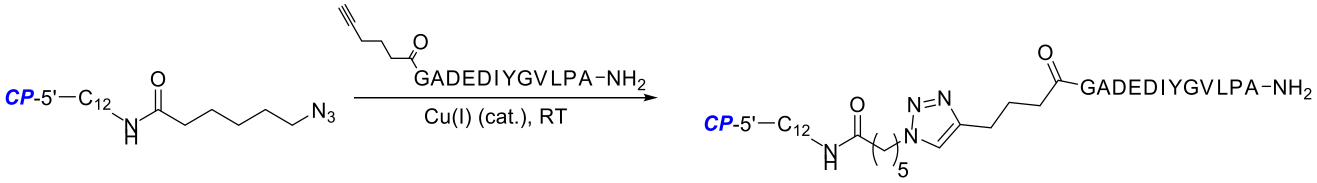

Synthesis of DNA-5′-LYNtide via CuAAC. 2nmol of CP-C_{12}-Cap(N_3) was suspended in a reaction mixture containing 2 M TEAA pH 6.5 (1.0 μL), 50 mM THPTA (4.0 μL), sat. CuBr in DMSO (2.0 μL of a fresh solution, premixed with THPTA), LYNtide Alkyne in DMSO (200 nmol, 100 eq.). The click reaction was performed by incubating for 3 hours at RT. 20 μL of 0.5 M EDTA, pH 8.0 was added to quench the reaction. The resulting conjugate was precipitated by adding 100μL of 10 M ammonium acetate and 800 μL of EtOH and being kept in -20°C overnight. After removal of supernatants, the resulting conjugate was purified by semi-prep HPLC at a flow rate of 1mL/min with H_2O (0.75% HFIP, 0.75%TEA, 5 μM EDTA, pH 7.0) and 10:90 H_2O:MeOH (0.75% HFIP, 0.75% TEA, 5μM EDTA, pH 7.0) as mobile phase. The resulting conjugate was characterized by ESI/MS. m/z: $[M-5H]^{5-}$, calcd. 1562.2, found 1562.9, $[M-6H]^{6-}$, calcd. 1301.7, found 1302.2, $[M-7H]^{7-}$, calcd. 1115.6, found 1116.1, $[M-8H]^{8-}$, calcd. 976.0, found 976.4, $[M-9H]^{9-}$, calcd. 867.4, found 867.9, $[M-10H]^{10-}$, calcd. 780.6, found 780.9, $[M-11H]^{11-}$, calcd. 709.5, found 709.9.

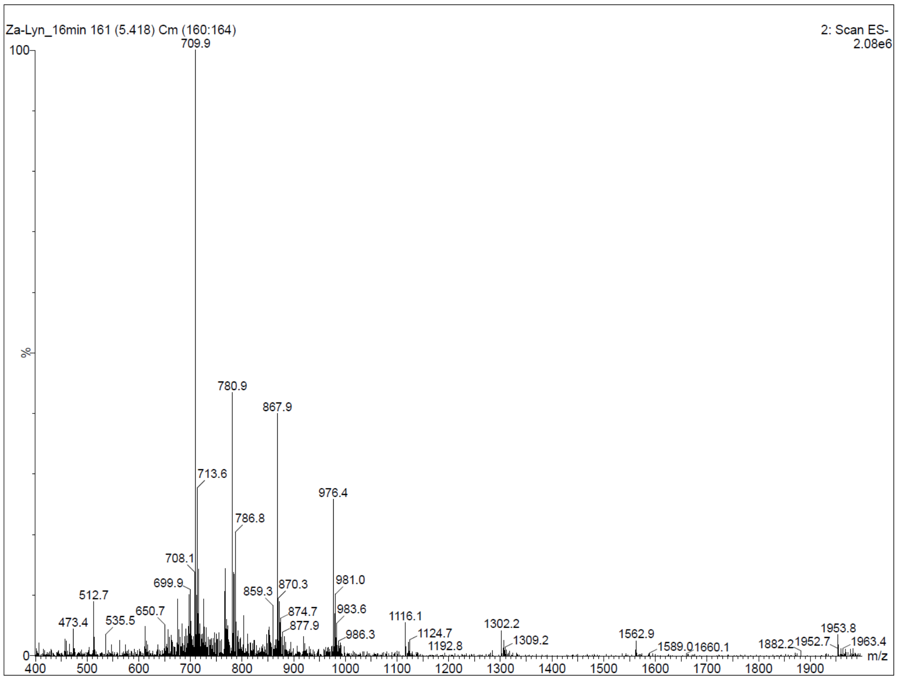

Synthesis of hit molecules off-DNA

Solid phase peptide and peptoid synthesis. Rink Amide MBHA resin (100 mg, 0.77 mmol/g) was swelled for 30 minutes at RT in dichloromethane, followed by incubation with 20% piperidine in DMF for 30 minutes. **a)** Couplings of Fmoc-amino acids were completed by double 30-minute incubations at RT with 5.0 eq. Fmoc amino acid, 5.0 eq. DIC and 5.0 eq. HOAt in DMF (premixed for 15 minutes). The resin was then incubated 30 minutes at RT with 20% piperidine for Fmoc removal. **b)** For acylations for peptoids monomers, 2 mL of 2 M bromoacetic acid (or 4-bromomethyl benzoic acid) was combined with 2 mL of 2 M DIC and pre-incubated for 10 minutes prior to an incubation with the resin for 30 minutes at 37°C. The resin was then incubated with 1 M amine in DMF for 1 hour at 37°C. Either a ninhydrin or chloranil test, as appropriate, was performed after each step to monitor all coupling, deprotection, and displacement reactions. The solid phase synthesis products were cleaved and deprotected in a cleavage cocktail containing 95% TFA, 2.5% TIPS and 2.5% H_2O for 3 hours at RT. The solvent was evaporated under Argon flow and then product precipitated by adding ice-cold diethyl ether. The precipitate was then purified by semi-prep HPLC at a flow rate of 5 mL/min with H_2O (0.1% TFA) and MeCN (0.1% TFA) as mobile phase. The purified compounds were characterized by ESI/MS or MALDI/MS.

*Synthesis of C-terminal tyramine peptide (**10-3**).* Aminomethyl ChemMatrix® resin (25 mg, 1.0 mmol/g) was swelled for 30 minutes at RT in MeOH. 4-(4-Formyl-3-methoxyphenoxy)-butyric acid (0.125 M in 0.8 mL DMF) and HOAt (0.025 M in 0.4mL MeOH) were mixed, combined with EDC·HCl (0.125 M in 0.8 mL MeOH), and incubated 30 minutes at RT with the resin. The resin was washed with 3x DMF and 3x MeOH, followed by addition of 5mL DMF and 5mL MeOH. To this, 10 eq. glacial HOAc was added with 10 eq. tyramine and 10 eq. sodium cyanoborohydride and gently stirred under light reflux at 80°C for 3 hours. The resin was drained, cooled, and washed with DMF, DCM, and MeOH and reswelled in MeOH. All following steps are as described in solid phase peptide and peptoid synthesis.

Compound characterization

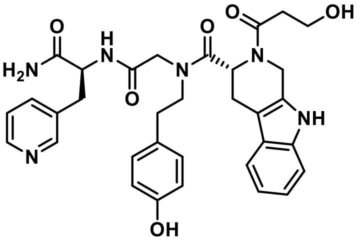

SrcDEL5

ESI/MS m/z: $[M+H]^+$ calcd. for $C_{33}H_{37}N_6O_6^+$, 613.3, found 613.3.

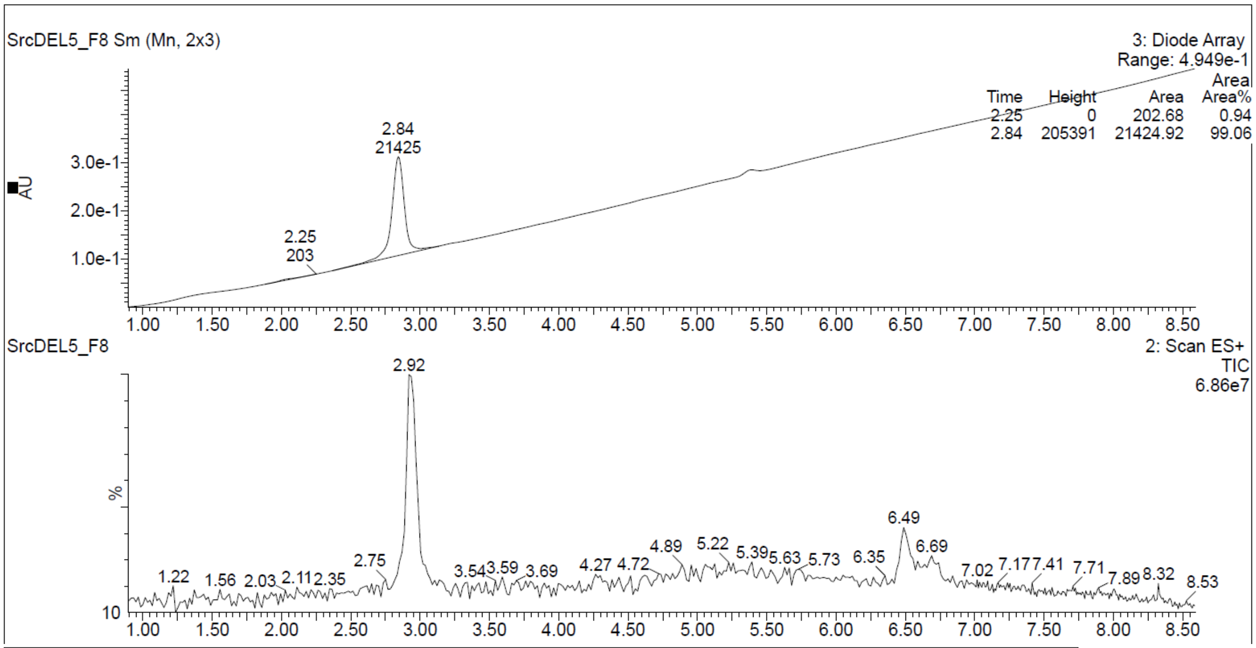

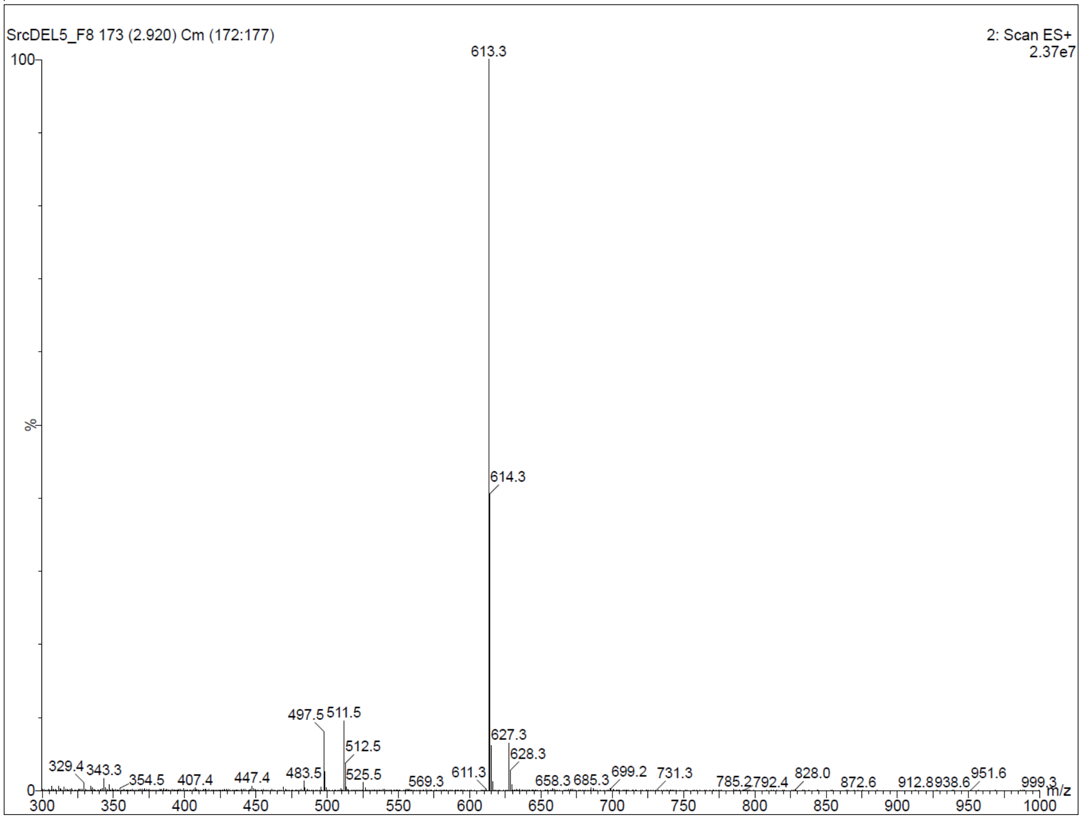

SrcDEL8

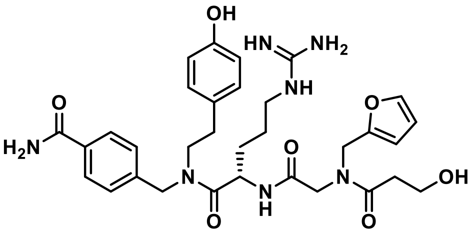

ESI/MS m/z: $[M+H]^+$ calcd. for $C_{32}H_{42}N_7O_7^+$, 636.3140, found 636.3058.

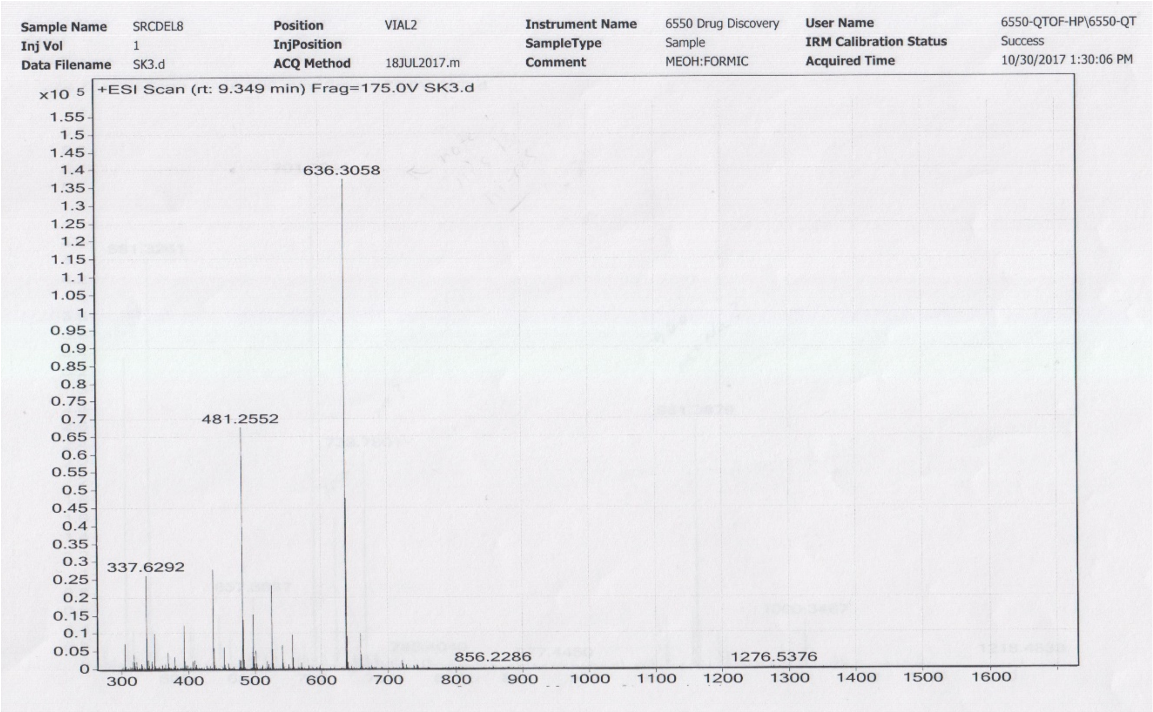

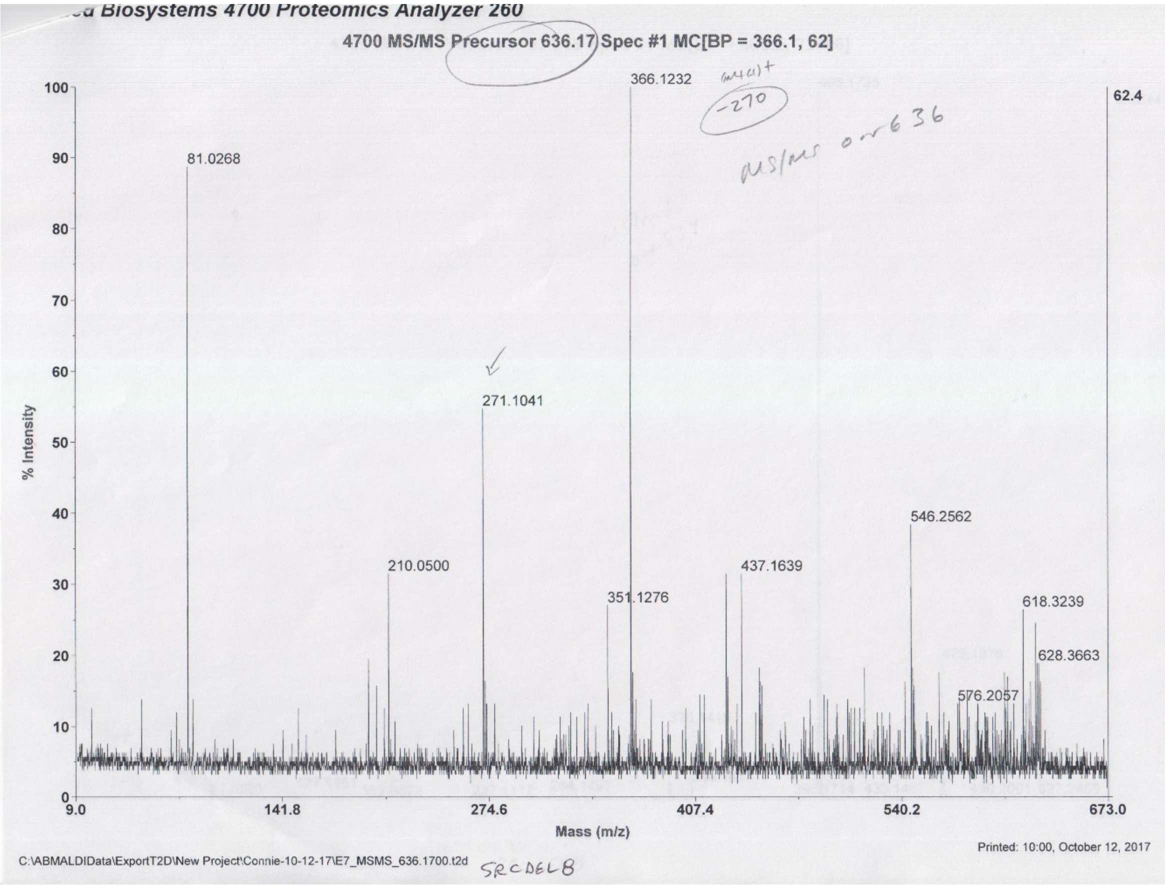

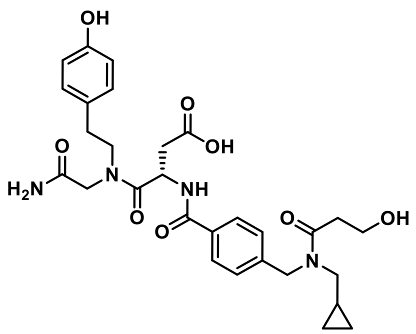

SrcDEL10

ESI/MS m/z: $[M+H]^+$ calcd. for $C_{29}H_{37}N_4O_8^+$, 569.3, found 569.4.

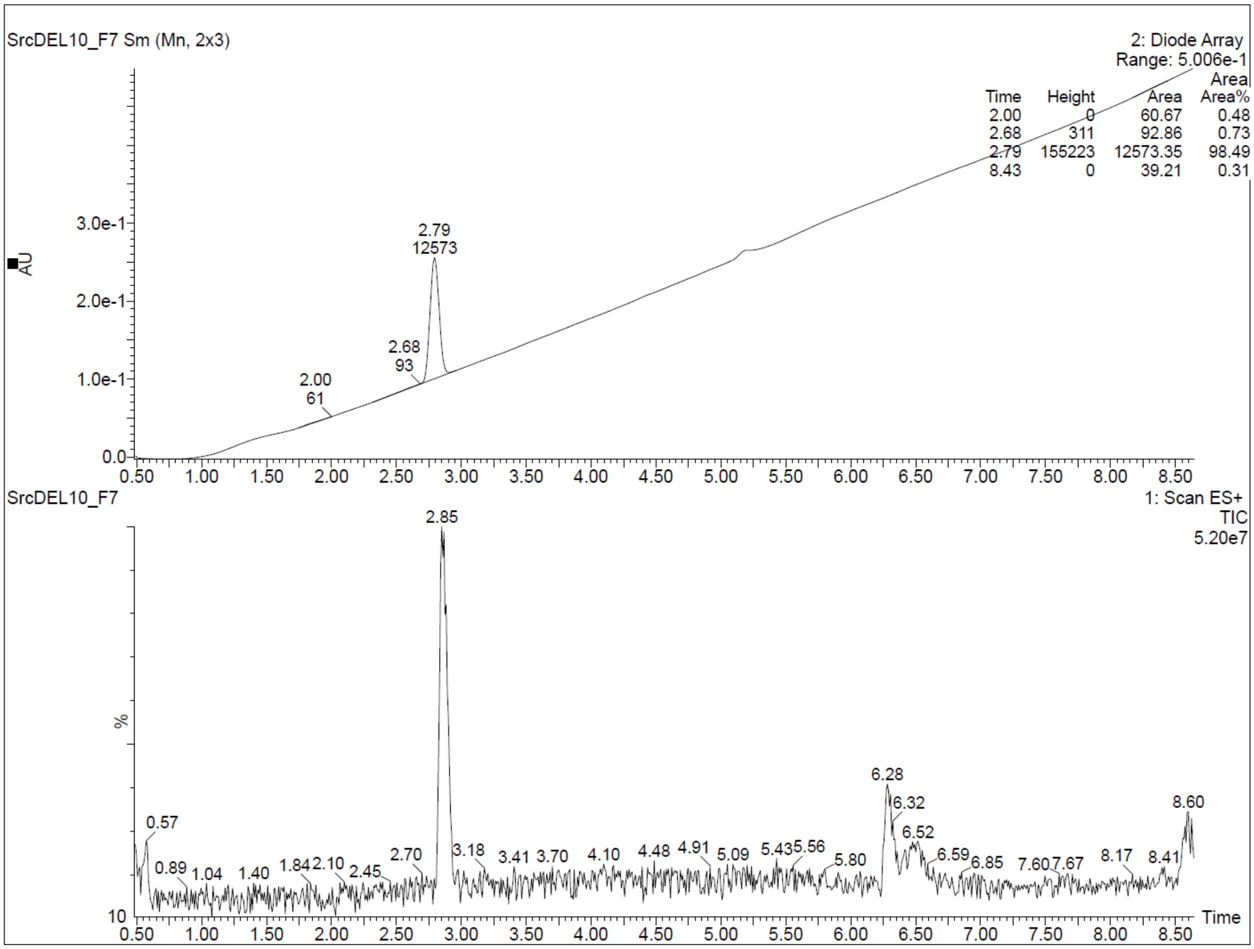

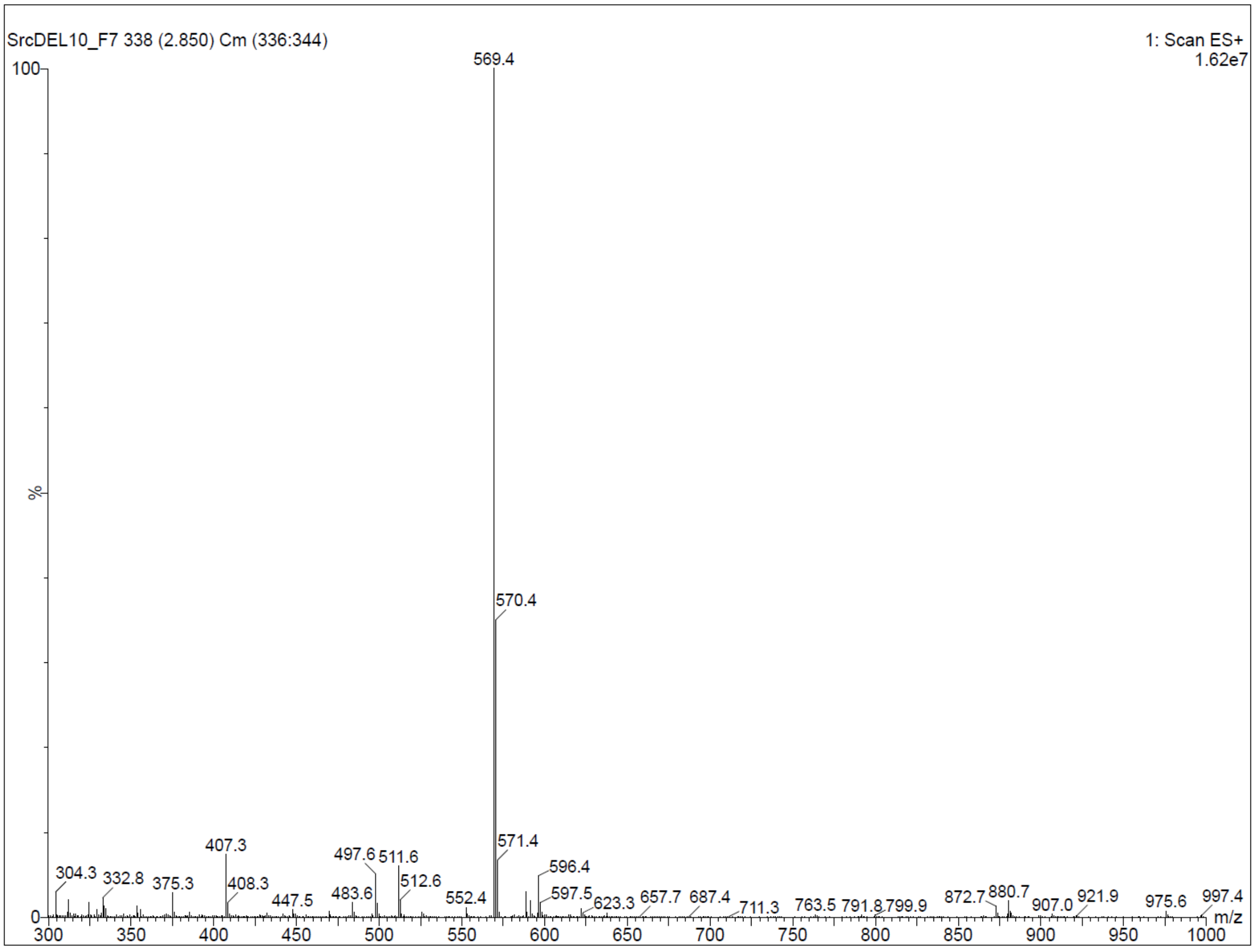
SrcDEL10_F7 338 (2.850) Cm (336:344)
1: Scan ES+
1.62e7
569.4
570.4
571.4
596.4
597.5
407.3
408.3
304.3
332.8
375.3
447.5
483.6
497.6
511.6
512.6
552.4
623.3
657.7
687.4
711.3
763.5
791.8
799.9
872.7
880.7
907.0
921.9
975.6
997.4
%
m/z

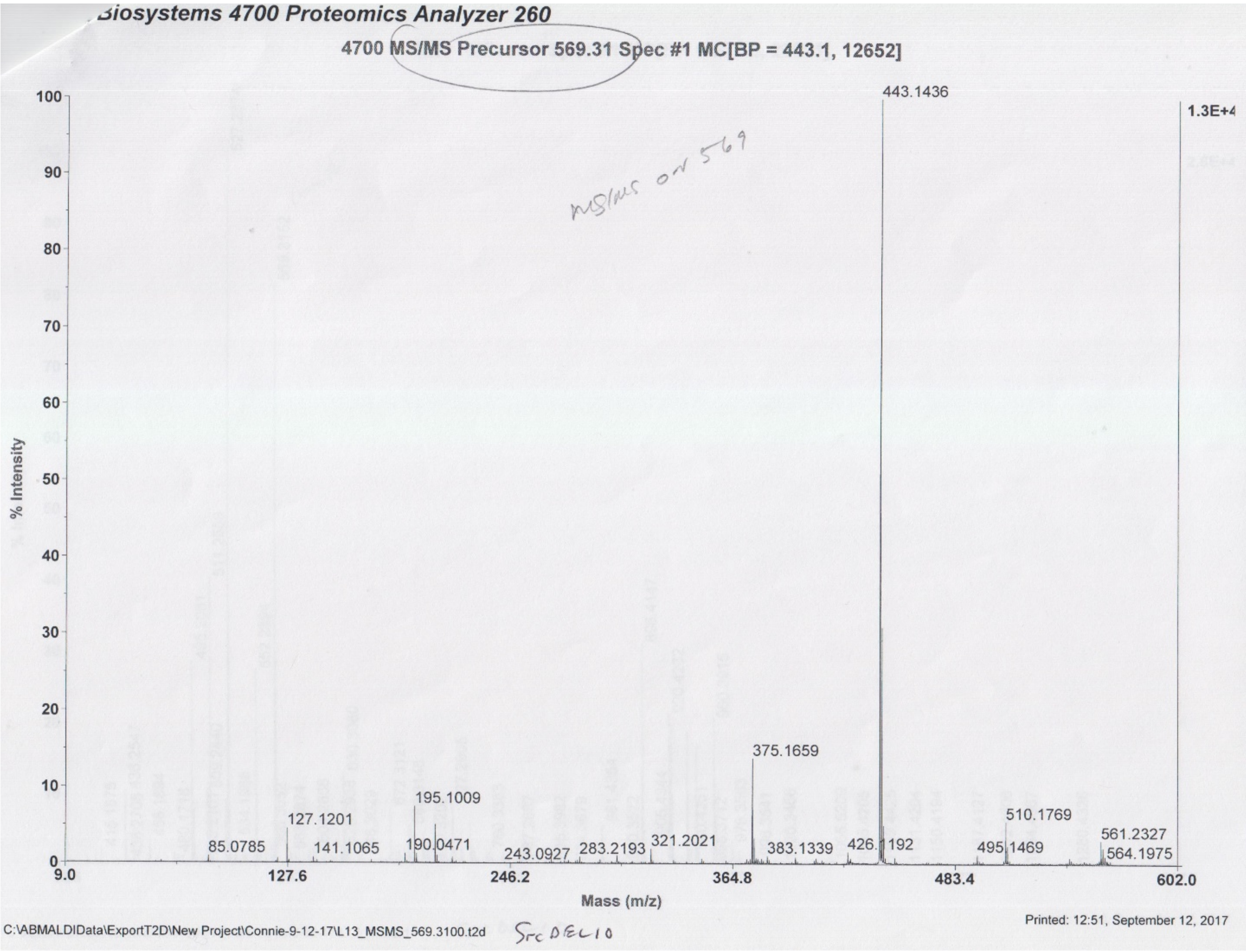
Biosystems 4700 Proteomics Analyzer 260
4700 MS/MS Precursor 569.31 Spec #1 MC[BP = 443.1, 12652]
443.1436
1.3E+4
MS/MS on 569
% Intensity
375.1659
195.1009
127.1201
85.0785
141.1065
190.0471
243.0927
283.2193
321.2021
383.1339
426.1192
495.1469
510.1769
561.2327
564.1975
Mass (m/z)
C:\ABMALDIData\ExportT2D\New Project\Connie-9-12-17\L13_MSMS_569.3100.t2d
SrcDEL10
Printed: 12:51, September 12, 2017

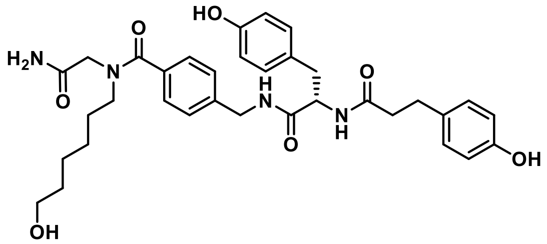

SrcDEL14

ESI/MS m/z: $[M+H]^+$ calcd. for $C_{34}H_{43}N_4O_7^+$, 619.3, found 619.4.

Crude LC/MS shown. Correct peak was HPLC purified prior to use.

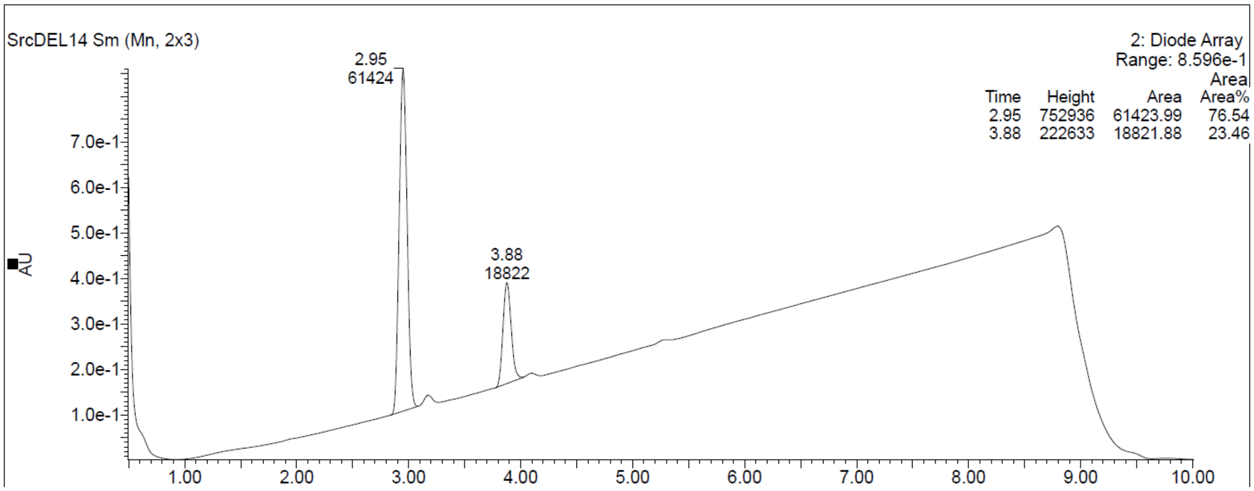

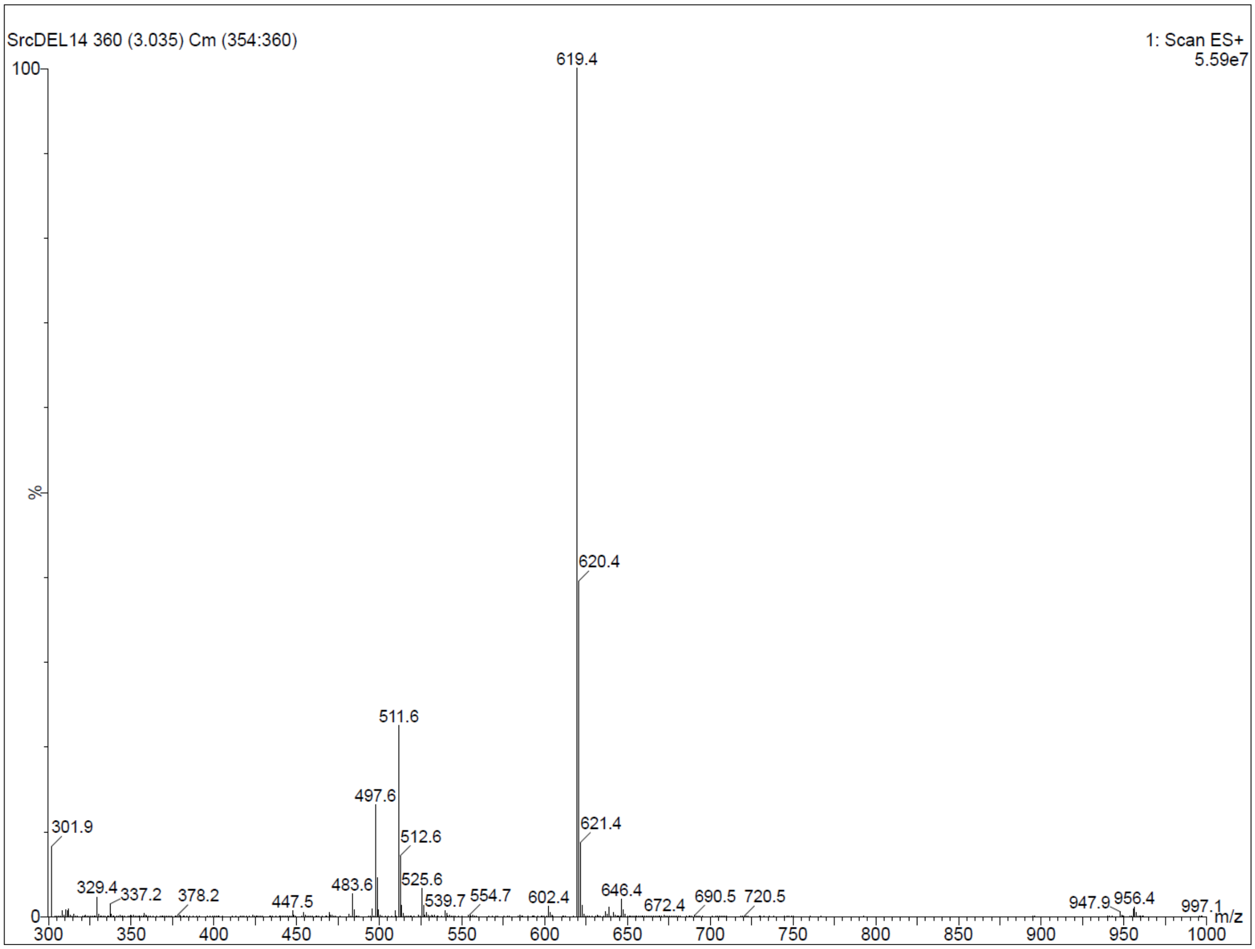

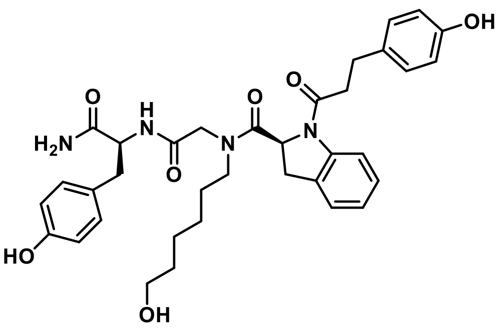

SrcDEL16

ESI/MS m/z: $[M+H]^+$ calcd. for $C_{35}H_{43}N_4O_7^+$, 631.3, found 631.4.

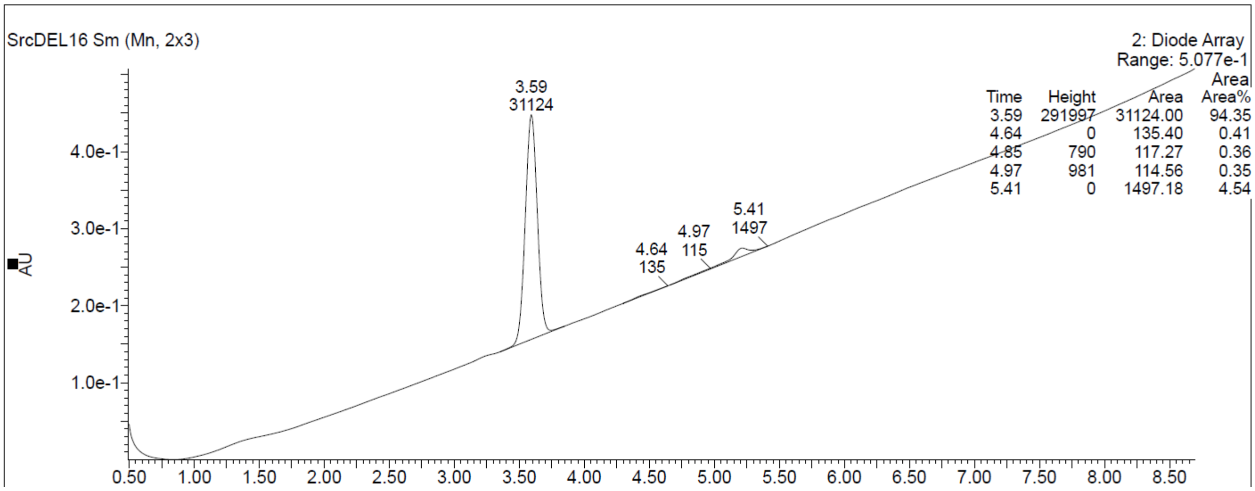

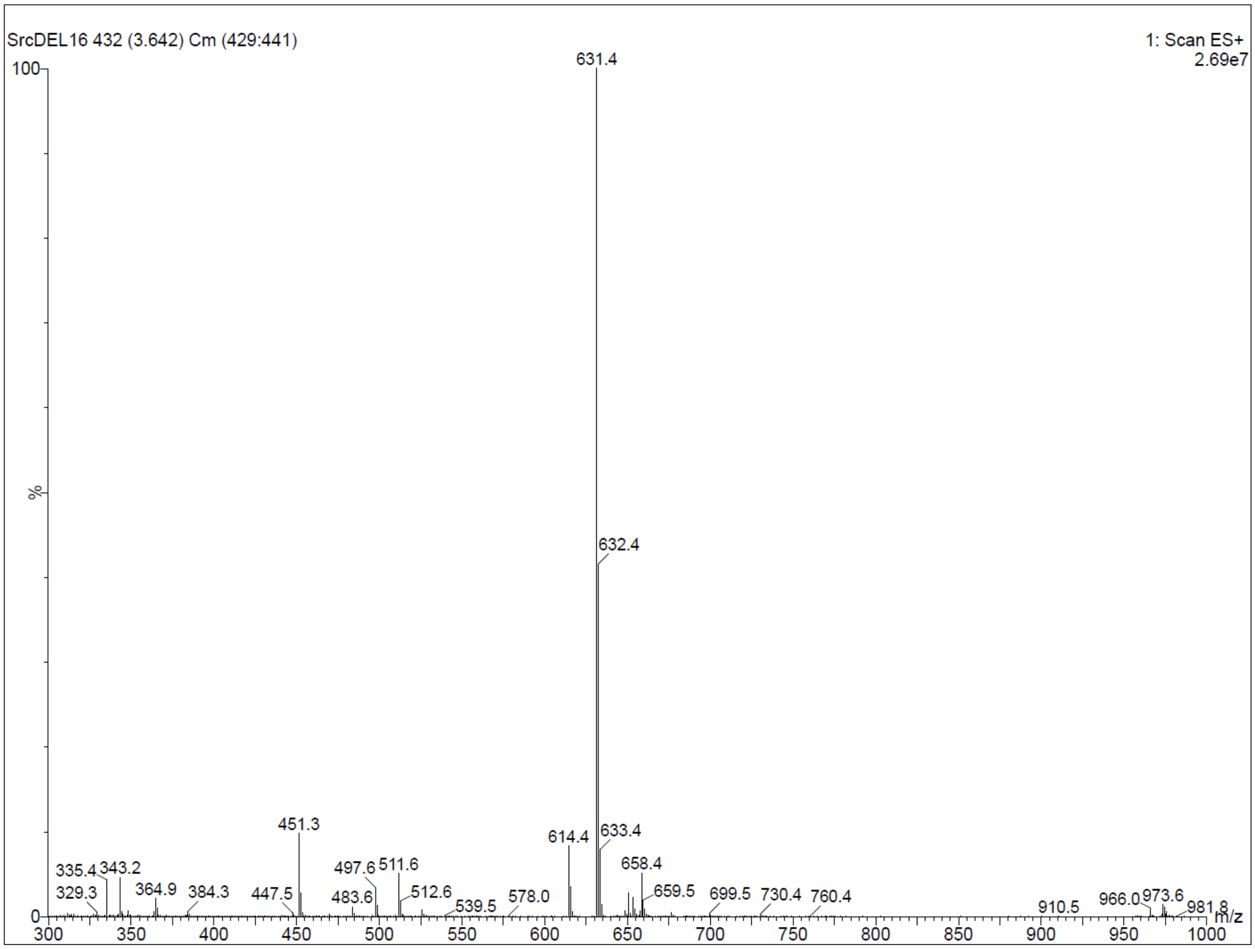

SrcDEL21 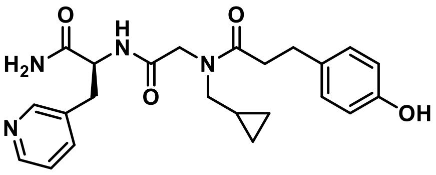

ESI/MS m/z: $[M+H]^+$ calcd. for $C_{23}H_{29}N_4O_4^+$, 425.2, found 425.3.

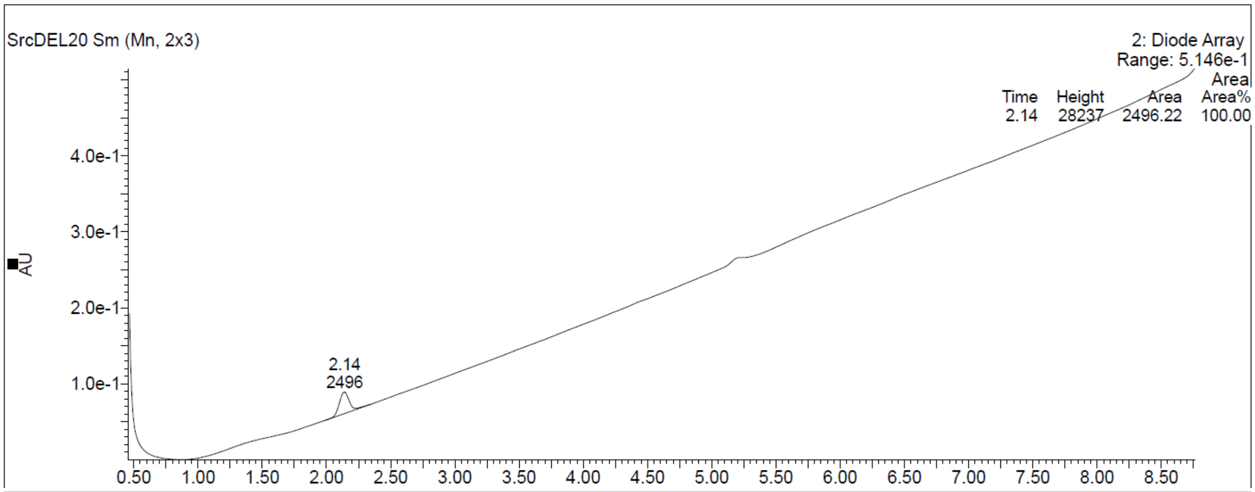

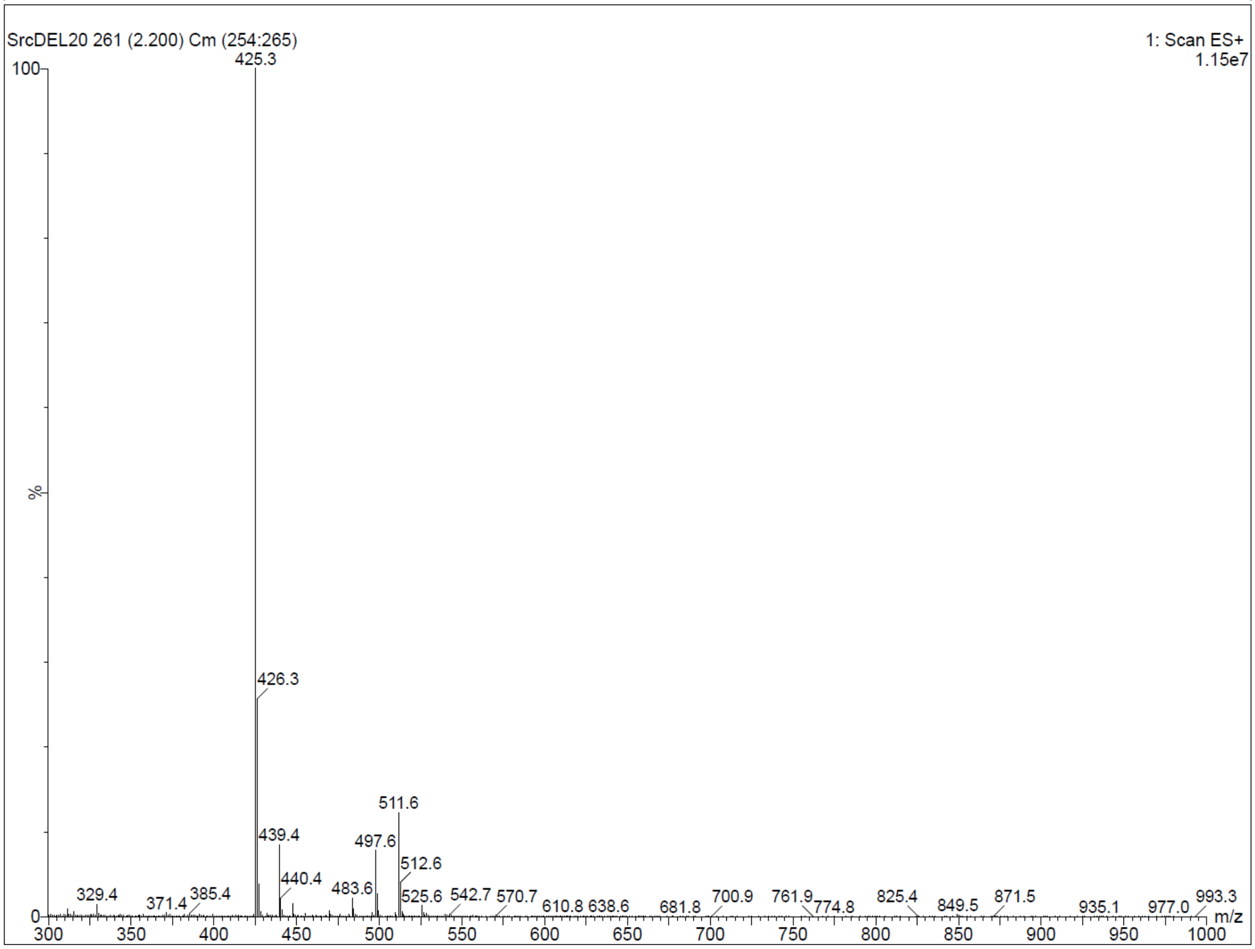

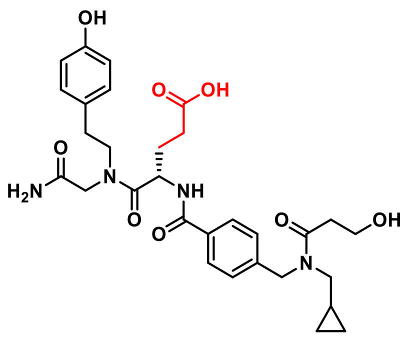

SrcDEL10-1

ESI/MS m/z: $[M+H]^+$ calcd. for $C_{30}H_{39}N_4O_8^+$, 583.3, found 583.3.

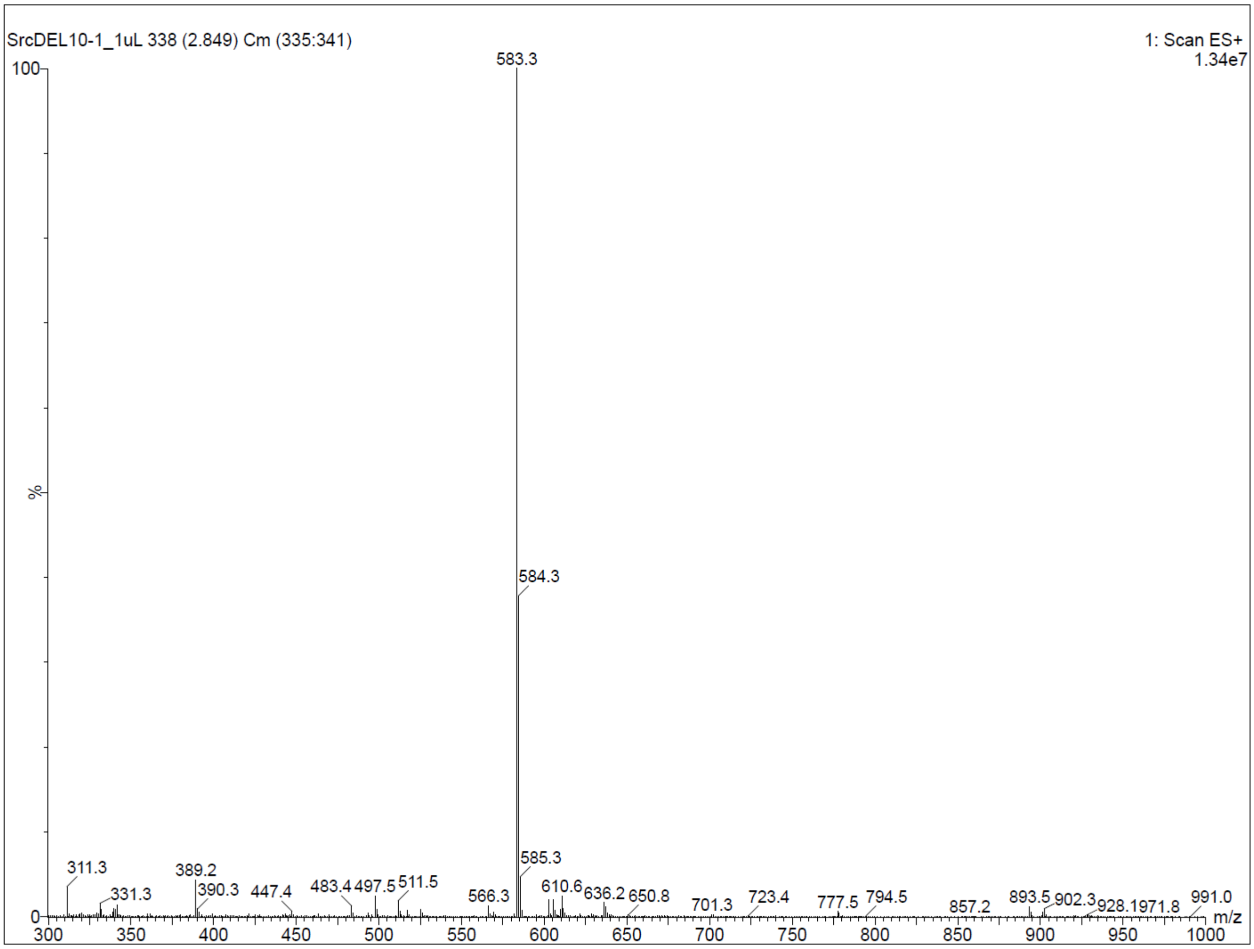

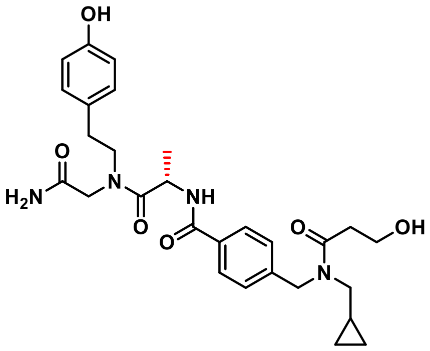

SrcDEL10-2

ESI/MS m/z: $[M+H]^+$ calcd. for $C_{28}H_{35}N_4O_8^+$, 555.2, found 555.3.

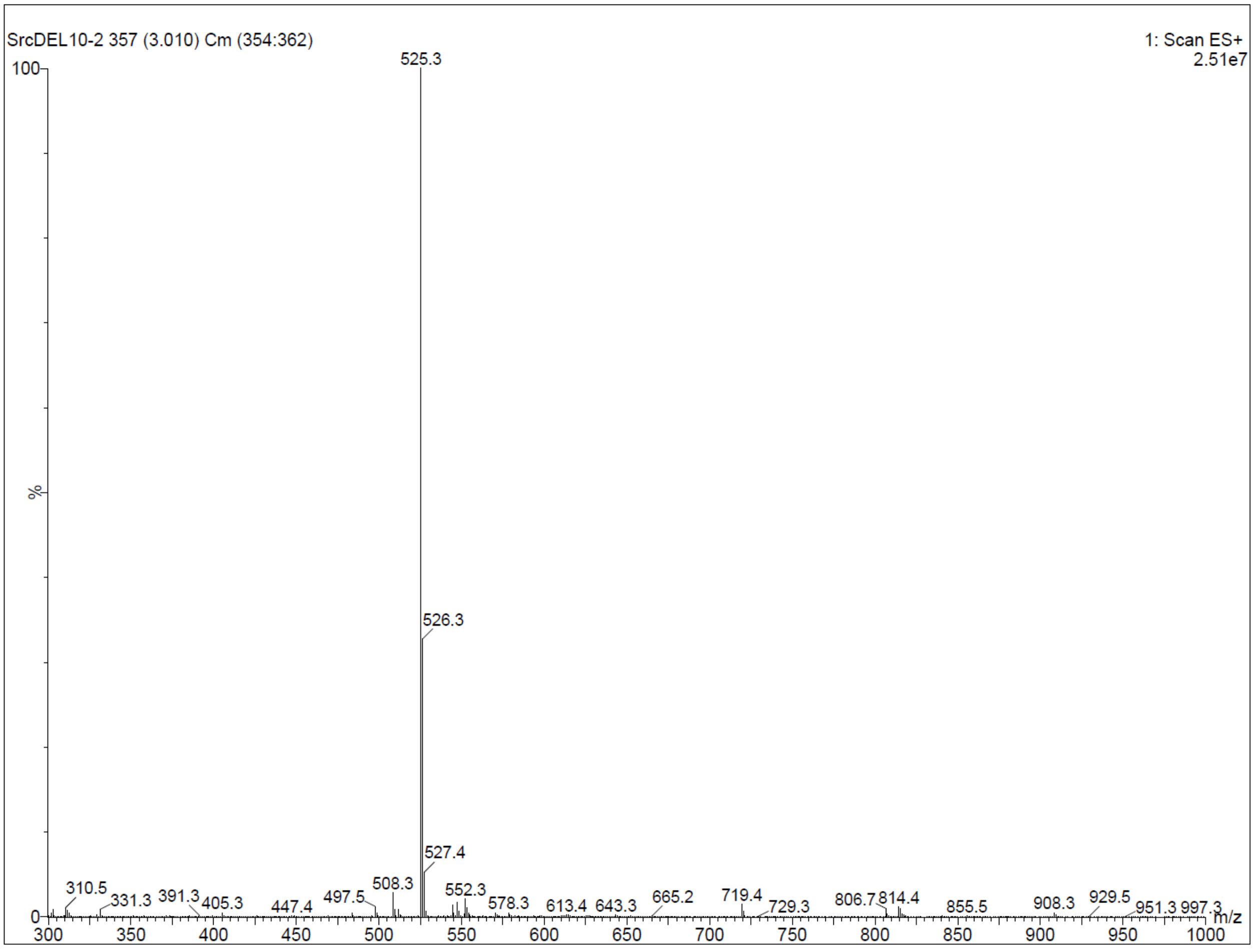

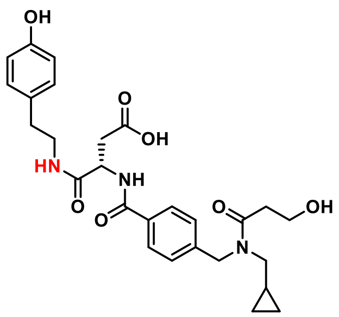

SrcDEL10-3

ESI/MS m/z: $[M+H]^+$ calcd. for $C_{27}H_{33}N_3O_7^+$, 512.24, found 512.46.

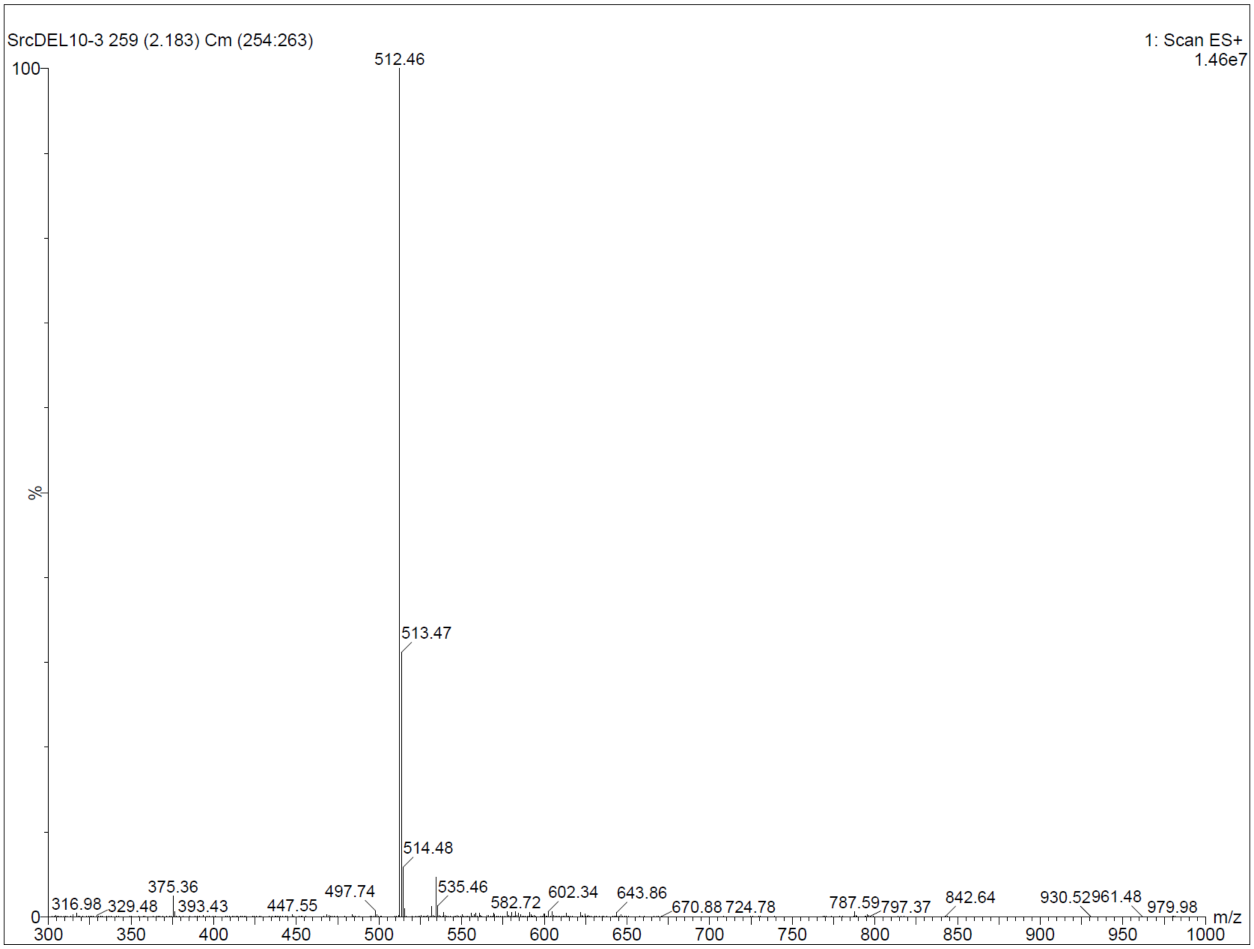

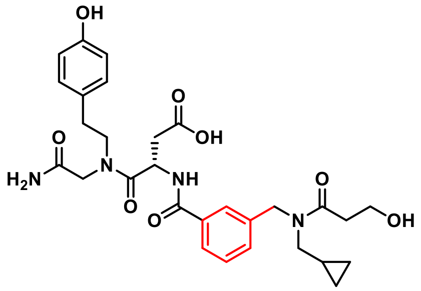

SrcDEL10-4

ESI/MS m/z: $[M+H]^+$ calcd. for $C_{29}H_{37}N_4O_8^+$, 569.3, found 569.3, $[M+Na]^+$, 591.3.

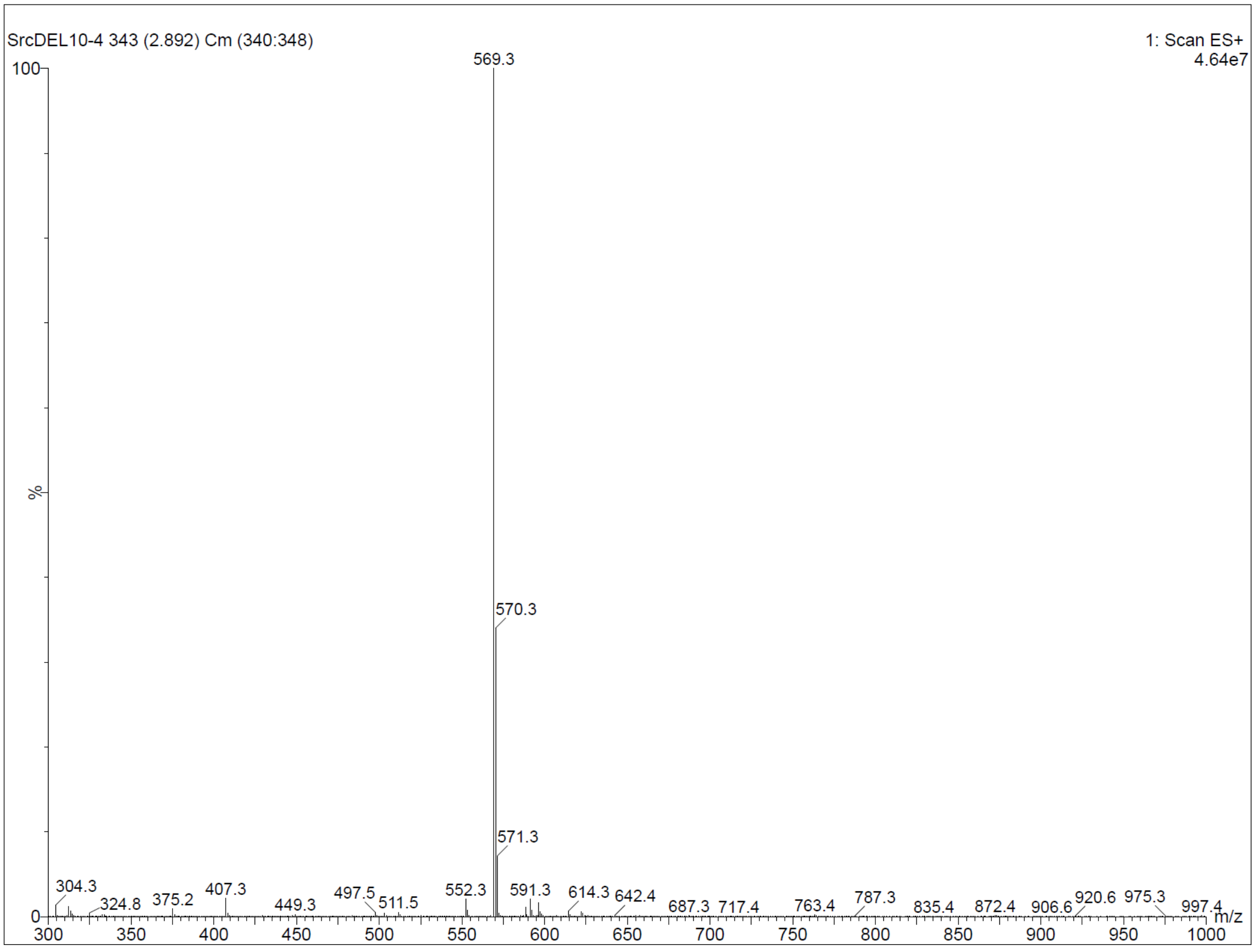

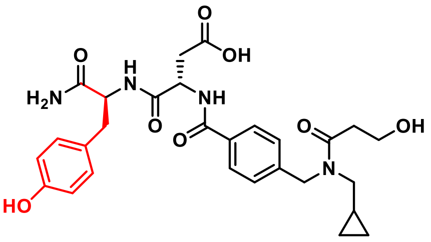

SrcDEL10-5

ESI/MS m/z: $[M+H]^+$ calcd. for $C_{28}H_{35}N_4O_8^+$, 555.2, found 555.3.

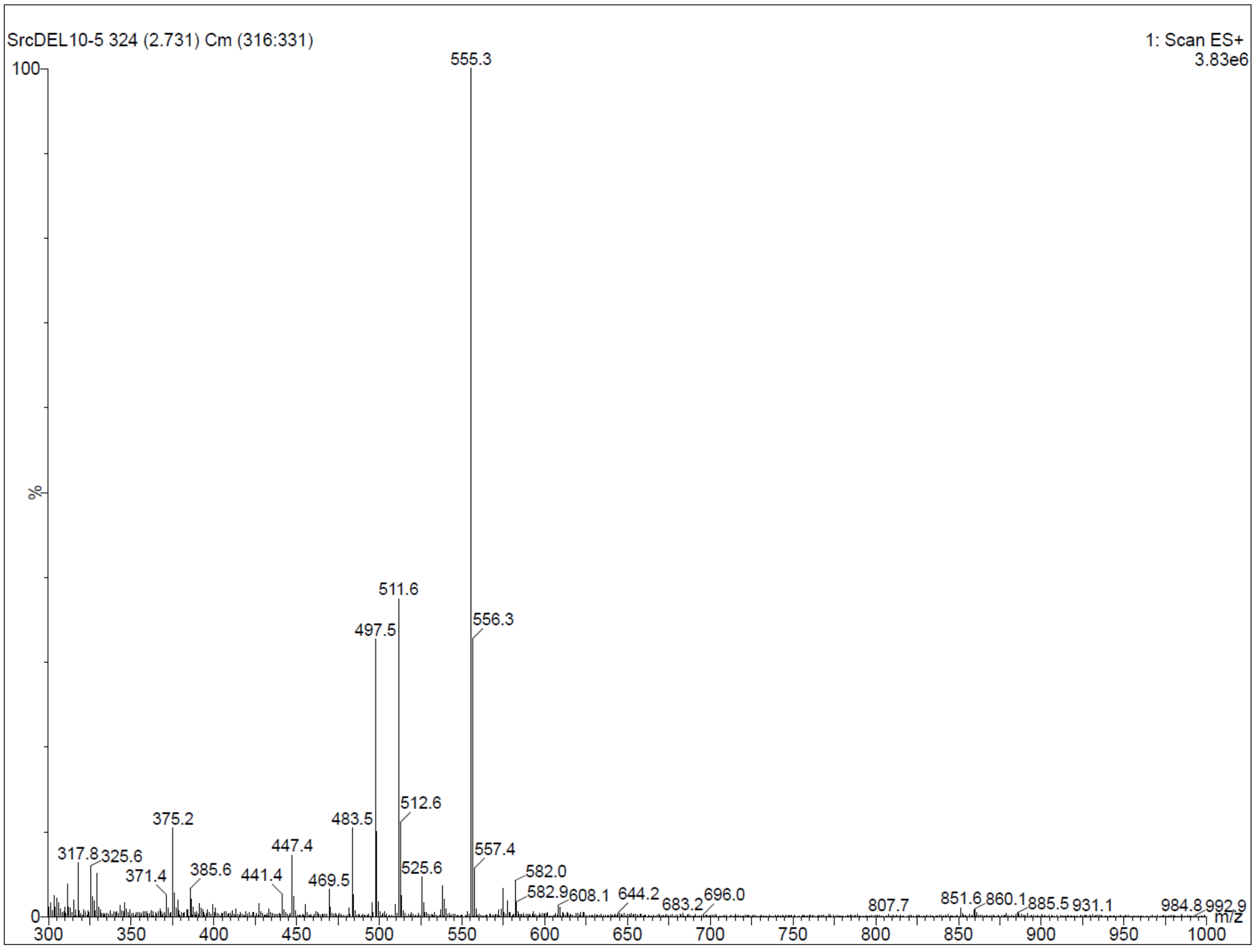

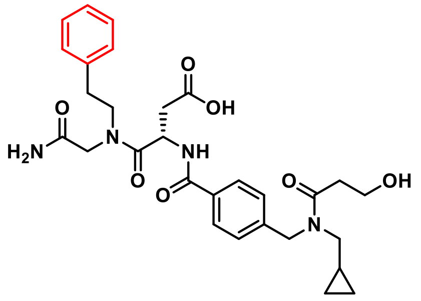

SrcDEL10-6

ESI/MS m/z: $[M+H]^+$ calcd. for $C_{29}H_{37}N_4O_7^+$, 553.27, found 553.49, $[M+Na]^+$, 575.48.

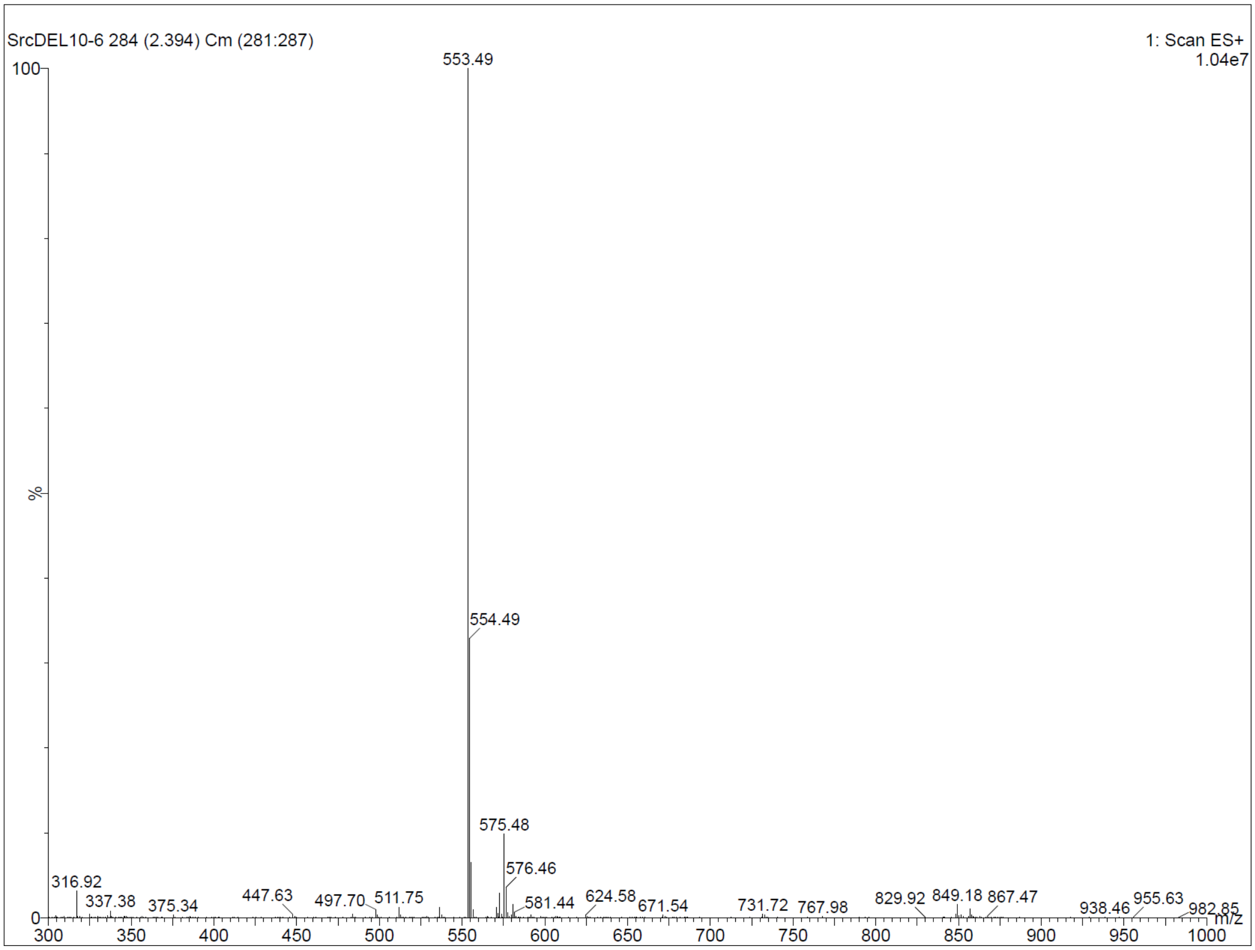

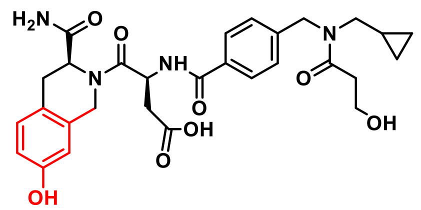

SrcDEL10-7

ESI/MS m/z: $[M+H]^+$ calcd. for $C_{29}H_{35}N_4O_8^+$, 567.2, found 567.3.

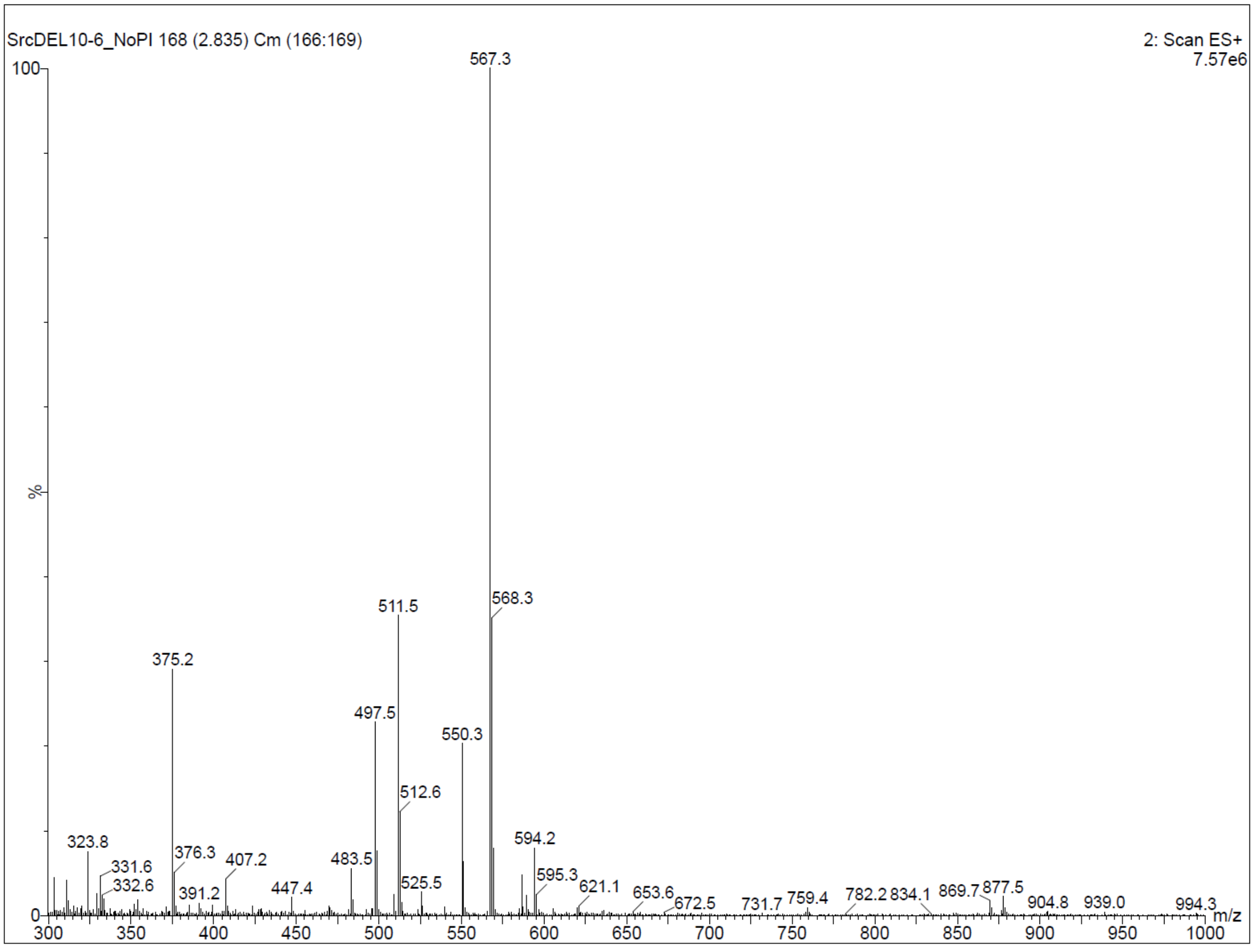

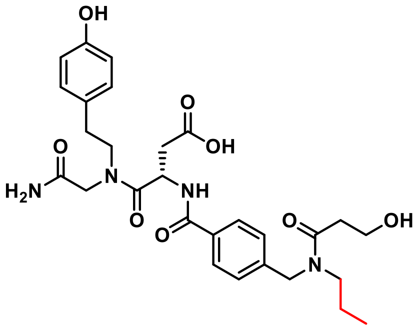

SrcDEL10-8

ESI/MS m/z: [M+H]+ calcd. for $C_{28}H_{37}N_4O_8^+$, 557.3, found 557.3, [M+Na]+, 579.3.

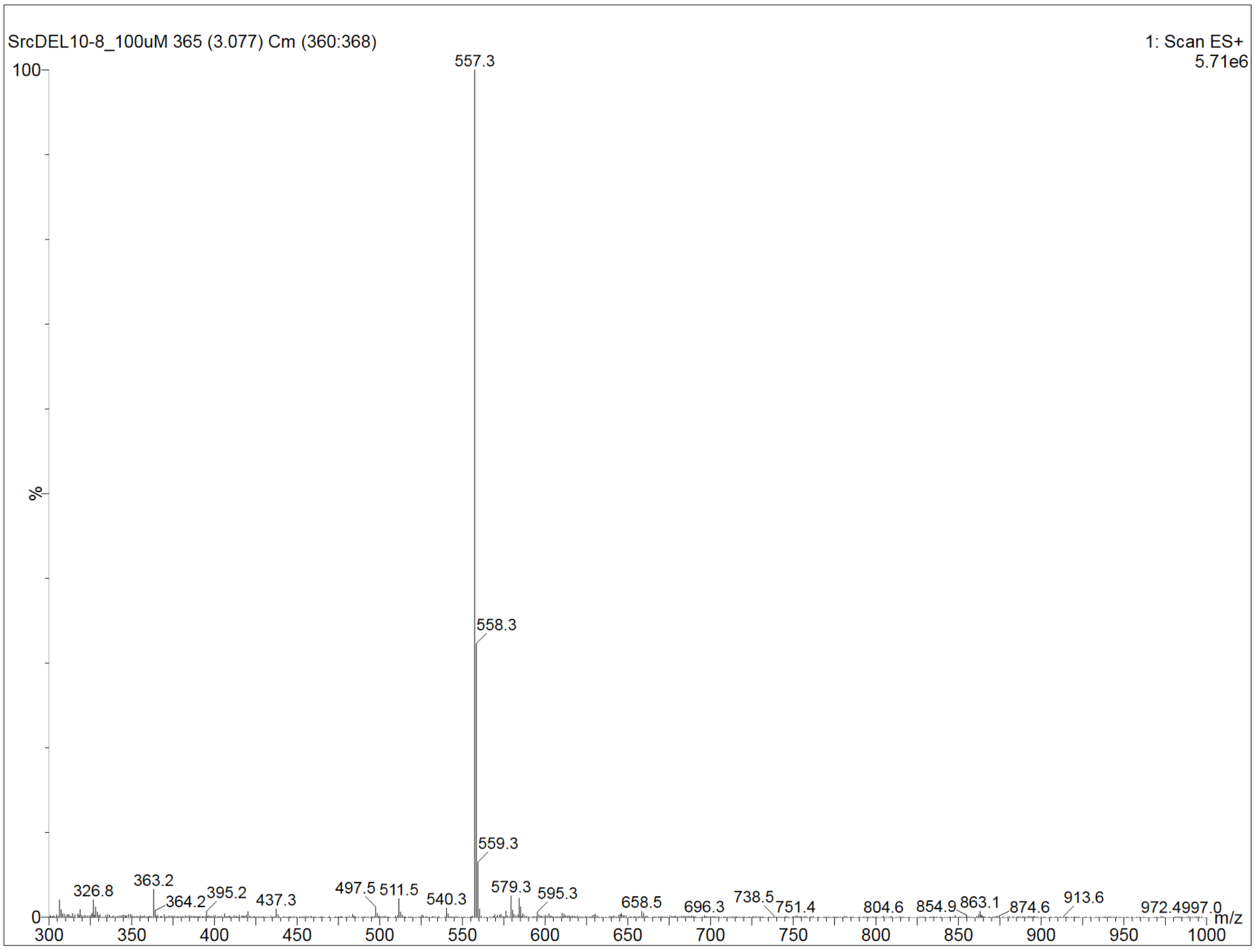

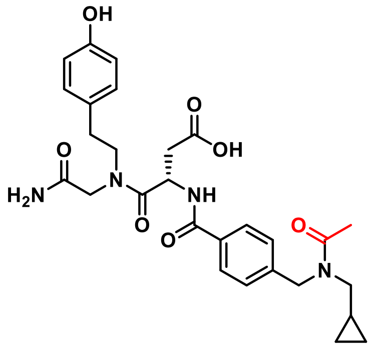

SrcDEL10-9

ESI/MS m/z: $[M+H]^+$ calcd. for $C_{28}H_{34}N_4O_7^+$, 539.25, found 539.48, $[M+Na]^+$, 561.48.

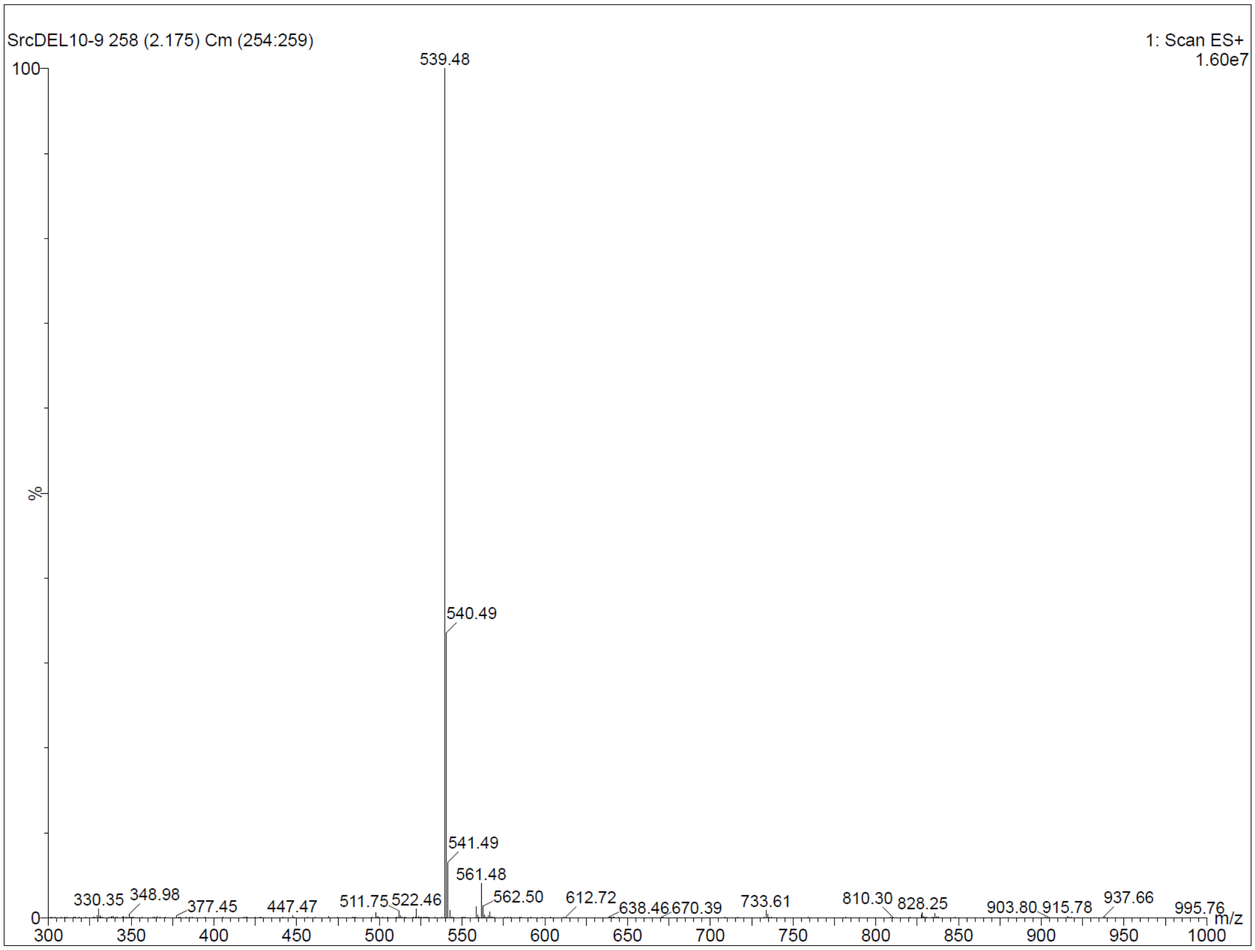

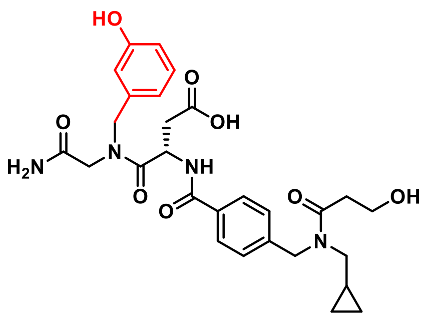

SrcDEL10-10

ESI/MS m/z: $[M+H]^+$ calcd. for $C_{28}H_{35}N_4O_8^+$, 583.28, found 555.48, $[M+Na]^+$, 577.48.

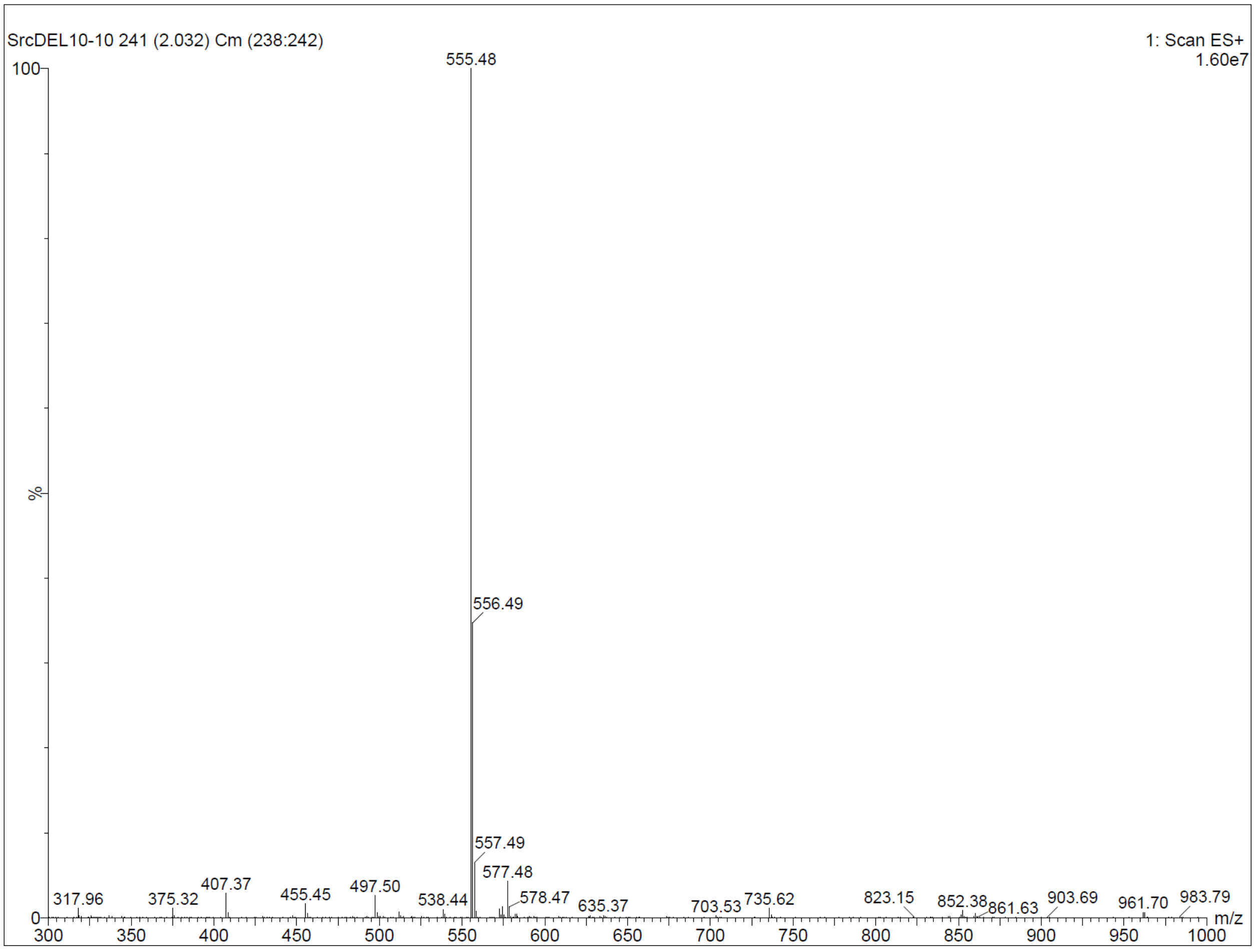

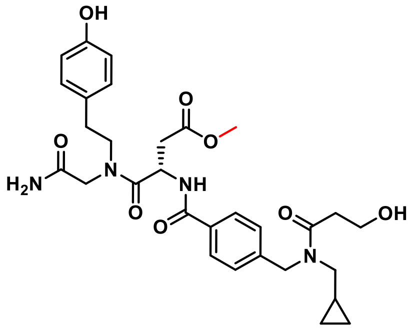

SrcDEL10-ester

ESI/MS m/z: $[M+H]^+$ calcd. for $C_{30}H_{39}N_4O_8^+$, 583.28, found 584.37, $[M+Na]^+$, 606.34.

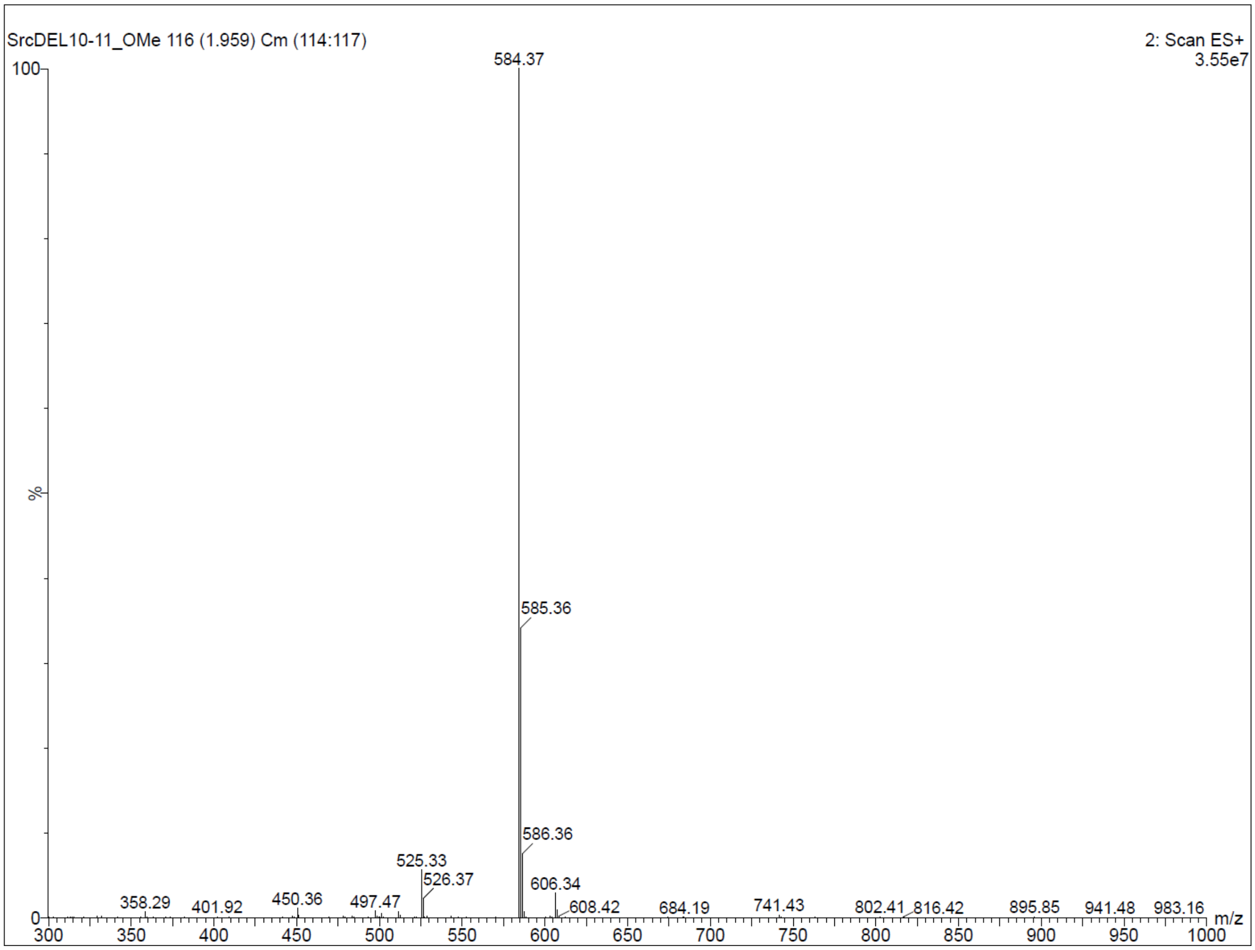

Supplementary References

1. Halpin, D.R.; Lee, J.A.; Wrenn, S.J.; Harbury, P.B. DNA display III. Solid-phase organic synthesis on unprotected DNA. *PLoS Biol.* **2004**, *2*, E175.
2. Denton, K.E.; Krusemark, C.J. Crosslinking of DNA-linked ligands to target proteins for enrichment from DNA-encoded libraries. *Med. Chem. Commun.* **2016**, *7*, 2020–2027.
3. Yim, C.-B.; Boerman, O.C.; de Visser, M.; de Jong, M.; Dechesne, A.C.; Rijkers, D.T.S.; Liskamp, R.M.J. Versatile conjugation of octreotide to dendrimers by cycloaddition (“click”) chemistry to yield high-affinity multivalent cyclic Peptide dendrimers. *Bioconjug. Chem.* **2009**, *20*, 1323–1331.
